# Interpenetration Phenomena via Anion Template Effects in Fe(II) and Co(II) Coordination Networks with a Bis-(1,2,4-triazole) Ligand

**DOI:** 10.3390/polym15153286

**Published:** 2023-08-03

**Authors:** Dustin N. Jordan, Patrick G. Straßburg, Dennis Woschko, Luca M. Carrella, Laure P. Cuignet, Katharina Eickmeier, Richard Dronskowski, Yann Garcia, Eva Rentschler, Christoph Janiak

**Affiliations:** 1 Institut für Anorganische Chemie und Strukturchemie, Heinrich-Heine-Universität, D-40204 Düsseldorf, Germany; dustin.jordan@uni-duesseldorf.de (D.N.J.); patrick.strassburg@uni-duesseldorf.de (P.G.S.); dennis.woschko@uni-duesseldorf.de (D.W.); laure.cuignet@sciensano.be (L.P.C.); 2 Department of Chemistry, Johannes Gutenberg University Mainz, D-55128 Mainz, Germany; carrella@uni-mainz.de (L.M.C.); rentschl@uni-mainz.de (E.R.); 3 Institute of Condensed Matter and Nanosciences, Molecular Chemistry, Materials and Catalysis (IMCN/MOST), Université Catholique de Louvain, 1348 Louvain-la-Neuve, Belgium; yann.garcia@uclouvain.be; 4 Institute of Inorganic Chemistry, RWTH Aachen University, D-52056 Aachen, Germany; katharina.eickmeier@rwth-aachen.de (K.E.); drons@hal9000.ac.rwth-aachen.de (R.D.)

**Keywords:** coordination polymers, coordination networks, metal–organic frameworks, triazole ligand, iron(II), cobalt(II), interpenetration, template effect, C-H⋯X hydrogen bonds, spin-crossover effect

## Abstract

Seven new coordination networks, [Fe(tbbt)_3_](BF_4_)_2_ (**1**), [Co(tbbt)_3_](BF_4_)_2_ (**2**), [Fe(tbbt)_3_](ClO_4_)_2_ (**3**), [Co(tbbt)_3_](ClO_4_)_2_ (**4**), [Fe(NCS)_2_(tbbt)_2_] (**5**), [Co(NCS)_2_(tbbt)_2_] (**6**), and [Fe(H_2_O)_2_(tbbt)_2_]Br_2_·2H_2_O (**7**), were synthesized with the linker 1,1’-(*trans*-2-butene-1,4-diyl)bis-1,2,4-triazole (tbbt) and structurally investigated. The structure of complexes **1**–**4** is composed of three interpenetrating, symmetry-related 3D networks. Each individual 3D network forms a primitive, nearly cubic lattice (**pcu**) with BF_4_^–^ or ClO_4_^–^ anions present in the interstitial spaces. The structure of compounds **5** and **6** is composed of two-dimensional **sql** layers, which are parallel to each other in the AB stacking type. These layers are interpenetrated by one-dimensional chains, both having the same formula unit, [M(NCS)_2_(tbbt)_2_] (M = Fe, Co). The structure of compound **7** consists of parallel, two-dimensional **sql** layers in the ABCD stacking type. The interpenetration in **1**–**6** is not controlled by π–π-interactions between the triazole rings or C=C bonds, as could have been expected, but by (triazole)C-H⋯F_4_B, C-H⋯O_4_Cl, and C-H⋯SCN anion hydrogen bonds, which suggests a template effect of the respective non-coordinated or coordinated anion for the interpenetration. In **7**, the (triazole)C-H⋯Br anion interactions are supplemented by O-H⋯O and O-H⋯Br hydrogen bonds involving the aqua ligand and crystal water molecules. It is evident that the coordinated and non-coordinated anions play an essential role in the formation of the networks and guide the interpenetration. All iron(II) coordination networks are colorless, off-white to yellow-orange, and have the metal ions in the high-spin state down to 77 K. Compound **5** stays in the high spin state even at temperatures down to 10 K.

## 1. Introduction

According to the IUPAC definition, coordination polymers consist of repeating coordination entities extending in one, two, or three dimensions [1,2,3]. Controlling their structure, thus leading to the desired properties for potential applications, can be described as one of the greatest goals of supramolecular chemistry in general [4,5]. Among the promising ways to achieve specific topologies and structures are self-assembly processes involving metal ions with different coordination geometries or radii and using counter anions or solvent molecules as templating agents [4,6,7,8]. While cations and anions as structure-guiding templates are well documented, a better understanding of their templating properties and their influence will eventually help to reach the ultimate aim of being able to control the structures and, therefore, the properties of the targeted products [8,9,10,11,12,13,14,15,16,17]. Examples for anion templating effects show that non-covalent interactions, like hydrogen-bonding, are involved [8,11,18,19].

The spin-crossover (SCO) phenomenon describes the transition from a low-spin (LS) to a high-spin (HS) state and vice versa of six-coordinated octahedral 3d^4^–3d^7^ metal ions induced by external stimuli like temperature, pressure, light irradiation, etc. [20,21,22,23,24,25,26,27,28]. Due to this entropy-driven electron redistribution through SCO, changes in chemical and physical properties like magnetism, color, structure, and dielectric behavior draws attention to potential applications such as switches, memory devices, sensors, or contrast agents [23,24,26,29,30,31,32,33,34,35]. Anions can play an important role in influencing these SCO properties [36,37,38,39].

Regarding SCO, iron(II) is by far the most studied metal ion concerning its property changes [23,26,40]. Most of these compounds have the d^6^-Fe^2+^ metal center surrounded by six nitrogen donor atoms, in which the metal–ligand bond lengths change by around 0.2 Å (10%) during spin transition [23,41]. SCO in cobalt(II) is less investigated, which could be ascribed to more gradual spin transitions [42,43,44]. Besides a bond length change by around 0.1 Å (5%), a significant Jahn–Teller distortion is expected for the LS state of an octahedrally coordinated d^7^-Co^2+^ metal ion [26,42,44,45,46].

1,2,4-triazole-based ligands are often used in coordination polymers and lead to interesting topologies, structures, and magnetic properties like SCO [5,47,48,49]. Previous studies show that the linker length can influence SCO properties and that, even with long ligands, SCO can be observed [43,50,51,52,53,54,55]. Here, we have synthesized Fe(II) and Co(II) coordination polymers with the bis-(1,2,4)-triazole ligand, 1,1′-(*trans*-2-butene-1,4-diyl)bis-1,2,4-triazole (tbbt), and different anions. The ligand tbbt was published first by Attaryan et al., and till now, there is only one mixed ligand structure known (Cd(tbbt)_2_(btc)_2_·2H_2_O, btc = 1,3,5-benzenetricarboxylate) [56,57]. Therefore, this work is a continuation of previously published results with different 1,1′-linked bis(1,2,4)-triazoles [47,48,58,59,60,61,62,63,64,65].

## 2. Materials and Methods

All chemicals were commercially obtained and used without further purification (see Supplementary Materials, Section S1). The water used was deionized.

FT-IR spectra were collected using a Bruker TENSOR 37 IR spectrometer (Bruker, Billerica, MA, USA) in ATR mode (platinum ATR-QL, diamond) in the range 4000–500 cm^−1^. NMR spectra were measured with a Bruker Avance III—300 (Bruker, Billerica, MA, USA) (^1^H: 300 MHz); ^13^C{^1^H}: 75 MHz). Elemental analyses were performed using a PerkinElmer 2400 series II elemental analyzer (PerkinElmer, Waltham, MA, USA) (accuracy of 0.5%). As coordination polymers cannot be recrystallized, any co-precipitate that cannot be removed by washing will affect the elemental analysis. It will be seen that out of the 8 × 3 = 24 CHN analysis values for the tbbt linker and the 7 complexes, 19 values lie within 0.5% deviation, 4 within 1%, and only one value deviates by 1.1% (C for compound 4). Thermogravimetric analyses were performed using a Netzsch TG209 F3 Tarsus (Netzsch, Selb, Germany) under a nitrogen atmosphere with a ramp of 5 K min^−1^ up to 600 °C. PXRD measurements were obtained with a Rigaku MiniFlex (Rigaku, Tokyo, Japan) (600 W, 40 kV, 15 mA) and Cu-K_α_ radiation (λ = 1.54184 Å) at room temperature. The increasing baseline below 5° 2Theta stems from the PXRD device measurement method with the low-background silicon holder. The highest reflex was normalized to 1. Simulated powder patterns were derived from the single-crystal data using MERCURY 2020.3.0 software [66].

Suitable single crystals were selected under a polarized-light Leica M80 microscope (Leica, Wetzlar, Germany) and mounted with oil on a cryo-loop. The data were obtained using a Rigaku XtaLAB Synergy S (Rigaku, Tokyo, Japan) diffractometer with a hybrid pixel array detector and a micro-focus sealed X-ray tube, PhotonJet (Cu) X-ray source (λ = 1.54184 Å). CRYSALISPRO was used for cell refinement, data reduction, and absorption correction [67]. The crystal structure was solved using OLEX2 with SHELXT, and the refinement was done with SHELXL [68,69,70]. Figures were drawn with DIAMOND 4.0 software [71]. The values for the distortion indices of the coordination polyhedra of **1**–**7** were calculated using OctaDist software [72]. For further information, see Supplementary Materials, Section S7.

SCO experiments were carried out as follows: single crystals of compounds **1**–**7** were selected and mounted with oil on a cryo-loop. The single crystals were cooled down to 80 K using the cryocooler of a Rigaku XtaLAB Synergy S diffractometer (Rigaku, Tokyo, Japan) at a rate of 0.5 K/min.

The temperature-dependent magnetic susceptibility of compound **5** was recorded using a Quantum Design SQUID magnetometer MPMS-XL7 (Quantum Design, San Diego, CA, USA). Measurements were made in a temperature range of 10 K to 300 K and under an applied external field of 0.1 Tesla. The temperature-dependent magnetic moments were corrected for the diamagnetic contribution of the holder, as well as for the diamagnetic contribution of the sample, which was determined using the Pascal constant.

For powder pressure experiments, powders of compounds **1**–**7** were pressed with a 30-ton press, a P30 hydraulic press by Research Industrial Instrument Co. (London, England), under vacuum in a 13 mm press tool for KBr pellets by the LOT-Oriel Group (LOT-Oriel Group, Darmstadt, Germany) at a pressure of 9 t for 10 min.

Diamond anvil cell (DAC) experiments were performed in a Boehler-Almax-DAC Diacell^®^ Bragg-LT(S) Plus (Almax easyLab Inc., Cambridge, MA, USA). Diamonds with 400 μm central tips and a tungsten carbide holder were used. The pressure inside the DAC was determined with the ruby fluorescence method. The corresponding ruby fluorescence spectrometer was based on the design by Y. Feng [73]. The light source was an LED with broadband emission around 565 nm. An Ocean Optics^®^ HR2000+ Raman spectrometer (Ocean Optics, Dunedin, FL, USA) was used for detection.

### 2.1. Synthesis

#### 2.1.1. Synthesis of 1,1′-(*trans*-2-butene-1,4-diyl)bis-1,2,4-triazole (tbbt)

The synthesis was carried out with a modified procedure from Shang et al. [74], as shown in Equation (1). Amounts of 4.0 g (57.9 mmol) of 1,2,4-triazole and 6.0 g (106.7 mmol) of KOH in 100 mL of acetonitrile were stirred for 30 min at 25 °C. A solution of 4.28 g (20.0 mmol) of *trans*-1,4-dibromo-2-butene in 60 mL of CH_3_CN was added, and the resulting mixture was stirred for a further 30 min at 25 °C. After filtration, the solvent was removed in vacuo. The resulting oil was dissolved in 30 mL of deionized H_2_O and washed with chloroform (4 × 50 mL). After the organic phase was dried with MgSO_4_ and filtrated, the excess of solvent was removed by rotary evaporation. The obtained yellowish product was purified by vacuum sublimation for 24 h at 140 °C. (Note: tbbt can also be used without purification by vacuum sublimation.) Yield: 1.93 g (51%). Before the analyses, the sample was dried in vacuo at 60 °C, as otherwise water signals will be detected. C_8_H_10_N_6_: calc. C = 50.5, H = 5.3, N = 44.2; exp.: C = 50.3, H = 5.4, N = 44.2. IR: ṽ [cm^−1^]: 2450, 3120, 3098, 3007, 1846, 1802, 1766, 1743, 1558, 1512, 1442, 1387, 1258, 1344, 1312, 1269, 1245, 1219, 1170, 1148, 1075, 1008, 977, 960, 929, 872, 758, 678, 642. ^1^H-NMR (300 MHz, DMSO-d^6^): δ [ppm]: 8.50 (s, 2H), 7.98 (s, 2H), 5.83–5.80 (m, 2H), 4.86–4.83 (q, 4H). ^13^C{^1^H}-NMR (75 MHz, DMSO-d^6^: δ [ppm]: 151.56, 143.95, 128.52, 49.60.

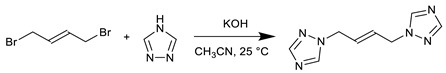
(1)

#### 2.1.2. Synthesis of [Fe(tbbt)_3_](BF_4_)_2_ (**1**) (Catena-bis(µ-1,1′-(trans-2-butene-1,4-diyl)bis-1,2,4-triazole-κ^2^N4,N4’)iron bis(tetrafluoridoborate))

The amounts of 61 mg (0.18 mmol) of Fe(BF_4_)_2_·6H_2_O and 103 mg (0.54 mmol) of tbbt were dissolved in 6 mL of H_2_O and stored at rt. After 24 h, colorless single crystals were obtained, washed three times with H_2_O (6 mL each), and stored in H_2_O. Yield: 82 mg (57%). C_24_H_30_B_2_F_8_FeN_18_: calc.: C = 36.0, H = 3.8, N = 31.5; exp.: C = 35.5, H = 3.8; N = 31.2. IR: ṽ [cm^−1^]: 3145, 2165, 1752, 1520, 1473, 1443, 1375, 1358, 1313, 1276, 1209, 1175, 1122, 1050, 1013, 990, 969, 941, 880, 759, 677, 652.

#### 2.1.3. Synthesis of [Co(tbbt)_3_](BF_4_)_2_ (**2**) (Catena-bis(µ-1,1′-(trans-2-butene-1,4-diyl)bis-1,2,4-triazole-κ^2^N4,N4’)cobalt bis(tetrafluoridoborate))

Compound **2** was synthesized similar to **1** from 61 mg (0.18 mmol) of Co(BF_4_)_2_·6H_2_O and 103 mg (0.54 mmol) of tbbt. Yield: 93.6 mg (65%). C_24_H_30_B_2_F_8_CoN_18_: calc.: C = 35.9, H = 4.1, N = 30.3; exp.: C = 35.4, H = 3.9, N = 31.2. IR: ṽ [cm^–1^]: 3148, 3029, 2965, 1751, 1520, 1474, 1443, 1375, 1359, 1313, 1278, 1209, 1174, 1121, 1050, 1013, 991, 969, 941, 880, 759, 677, 652.

#### 2.1.4. Synthesis of [Fe(tbbt)_3_](ClO_4_)_2_ (**3**) (Catena-bis(µ-1,1′-(trans-2-butene-1,4-diyl)bis-1,2,4-triazole-κ^2^N4,N4′)iron bis(perchlorate))

*Please note that perchlorates are potentially explosive and must be handled with care.* TGA shows an explosive decomposition at around 250 °C. In total, 46 mg (0.18 mmol) of Fe(ClO_4_)_2_·xH_2_O and 103 mg (0.54 mmol) of tbbt were dissolved in 6 mL of H_2_O and stored at rt. After 24 h, yellow single crystals were obtained, washed three times with H_2_O (6 mL each), and stored in H_2_O. Yield: 66 mg (44%). C_24_H_30_Cl_2_O_8_FeN_18_: calc.: C = 34.9, H = 3.7, N = 30.6; exp.: C = 34.1, H = 3.8, N = 29.9. IR: ṽ [cm^−1^]: 3144, 2960, 2164, 2018, 1751, 1518, 1472, 1439, 1373, 1358, 1311, 1276, 1208, 1172, 1121, 1090, 1013, 989, 968, 939, 879, 759, 676, 651, 622.

#### 2.1.5. Synthesis of [Co(tbbt)_3_](ClO_4_)_2_ (**4**) (Catena-bis(µ-1,1′-(trans-2-butene-1,4-diyl)bis-1,2,4-triazole-κ^2^N4,N4’)cobalt bis(perchlorate))

*Please note that perchlorates are potentially explosive and should be handled with care.* TGA shows an explosive decomposition at around 340 °C. In total, 33 mg (0.09 mmol) of Co(ClO_4_)_2_·6H_2_O was dissolved in 2 mL of H_2_O, while 35 mg (0.18 mmol) of tbbt was dissolved in 5 mL of H_2_O. Both solutions were heated to their boiling points, combined, and cooled to rt at ambient conditions. Orange crystals were obtained overnight, washed three times with H_2_O (7 mL each), and stored in H_2_O. Yield: 34 mg (46%). C_24_H_30_Cl_2_O_8_CoN_18_: calc.: C = 34.8, H = 3.7, N = 30.4; exp.: C = 33.7, H = 3.7, N = 29.8. The jointly lower C and N analysis values may indicate the formation of a very small amount of cobalt(hydroxide)oxide co-precipitate. IR: ṽ [cm^−1^]: 3146, 2960, 1749, 1519, 1472, 1440, 1373, 1312, 1277, 1208, 1172, 1091, 1077, 1013, 990, 968, 938, 878, 758, 677, 651, 622, 475.

#### 2.1.6. Synthesis of [Fe(NCS)_2_(tbbt)_2_] (**5**) (Catena-bis(thiocyanato-κN)-bis(µ-1,1’-(trans-2-butene-1,4-diyl)bis-1,2,4-triazole-κ^2^N4,N4’)iron)

Compound **5** was synthesized according to Garcia et al. [75]. A solution of 90 mg (0.23 mmol) of (NH_4_)_2_Fe(SO_4_)_2_·6H_2_O and 51 mg (0.29 mmol) of ascorbic acid in 1.25 mL of H_2_O was heated to near its boiling point and added to a hot solution of 35 mg (0.46 mmol) of NH_4_SCN in 1 mL of H_2_O. To this, a nearly boiling solution of 88 mg (0.46 mmol) of tbbt in 5 mL of H_2_O was added dropwise. This final hot solution was cooled to rt within two days. The colorless crystals were washed with H_2_O three times (6 mL each) and were stored in H_2_O. Yield: 74 mg (58%). C_18_H_20_S_2_FeN_14_: calc.: C = 39.1, H = 3.7, N = 35.5, S = 11.6; exp.: C = 38.9, H = 3.7, N = 35.5, S = 11.9. IR: ṽ [cm^−1^]: 3144, 3104, 3026, 2949, 2867, 2847, 2071, 2050, 1822, 1792, 1770, 1734, 1690, 1518, 1470, 1439, 1426, 1391, 1377, 1339, 1318, 1278, 1206, 1182, 1127, 1075, 1033, 1013, 986, 969, 949, 899, 888, 868, 848, 788, 760, 698, 675, 643, 582.

#### 2.1.7. Synthesis of [Co(NCS)_2_(tbbt)_2_] (**6**) (Catena-bis(thiocyanato-κN)-bis(µ-1,1′-(trans-2-butene-1,4-diyl)bis-1,2,4-triazole-κ^2^N4,N4’)cobalt)

The amount of 16 mg (0.09 mmol) of Co(SCN)_2_ was dissolved in 2 mL of H_2_O, while 35 mg (0.18 mmol) of tbbt was dissolved in 5 mL of H_2_O. Both solutions were heated to nearly their boiling points, combined, and cooled to rt over 48 h. The obtained orange crystals were washed three times with H_2_O (7 mL each) and stored in H_2_O. Yield: 36 mg (36%). C_18_H_20_S_2_CoN_14_: calc.: C = 38.9, H = 3.6, N = 35.3, S = 11.5; exp.: C = 38.5, H = 3.8, N = 35.2, S = 11.9. IR: ṽ [cm^−1^]: 3145, 3105, 2949, 2852, 2081, 2058, 2015, 1825, 1792, 1770, 1734, 1689, 1519, 1471, 1440, 1425, 1392, 1378, 1339, 1319, 1279, 1205, 1177, 1127, 1069, 1034, 1014, 1007, 988, 970, 948, 916, 901, 889, 868, 847, 788, 760, 698, 675, 643, 582, 480, 467, 423.

#### 2.1.8. Synthesis of [Fe(H_2_O)_2_(tbbt)_2_]Br_2_·2H_2_O (**7**) (Catena-diaqua-bis(µ-1,1′-(trans-2-butene-1,4-diyl)bis-1,2,4-triazole-κ^2^N4,N4’)iron Dibromide Dihydrate)

Compound **7** was synthesized similar to Lavrenova et al. [76]. In total, 47 mg (0.25 mmol) of FeSO_4_·2H_2_O and 10 mg (0.06 mmol) of ascorbic acid were dissolved in 3 mL of H_2_O, to which a solution of 66 mg (0.25 mmol) of Ba(NO_3_)_2_ in 3 mL of H_2_O was added. The mixture was left for 2 h at rt; then, the BaSO_4_ precipitate was filtered off, and 120 mg (1 mmol) of KBr was added to this solution, which was heated to nearly boiling and added to a nearly boiling solution of 95 mg (0.50 mmol) of tbbt in 3 mL of EtOH. The combined hot solution was cooled to rt at ambient conditions, and colorless crystals were obtained overnight. The crystals were washed with H_2_O three times (6 mL each) and stored in H_2_O. Yield: 131 mg (79%). C_16_H_20_FeN_12_Br_2_(H_2_O)_2_·2H_2_O: calc.: C = 28.8, H = 4.2, N = 25.2; exp.: C = 28.4, H = 4.3, N = 25.2. IR: ṽ [cm^−1^]: 3472, 3386, 3257, 3144, 3096, 2968, 2941, 2075, 1812, 1731, 1620, 1583, 1526, 1476, 1441, 1383, 1369, 1345, 1311, 1276, 1209, 1181, 1132, 1098, 1027, 1005, 980, 908, 880, 866, 776, 756, 707, 676, 655, 642, 574, 528, 486, 423.

## 3. Results and Discussion

The linker 1,1′-(*trans*-2-butene-1,4-diyl)bis-1,2,4-triazole (tbbt) was obtained from the reaction between 1,2,4-triazole and *trans*-1,4-dibromo-2-butene in acetonitrile, as depicted in Equation (1), and its identity was established by ^1^H, ^13^C NMR and IR spectroscopy. Thermogravimetric analysis indicates a stability of tbbt of up to 200 °C (see Supplementary Materials, Section S2).

**Scheme 1:**
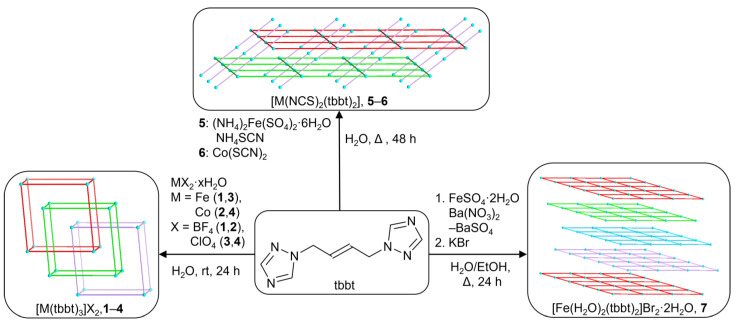
Schematic presentation of the synthesis of the coordination networks **1**–**7** with their formed structure topologies. The blue spheres represent the metal atoms and the different colors of the connecting lines differentiate the adjacent networks. The *anti*-conformeric tbbt linkers lie along the colored connecting lines except for the for the pink chains in **5** and **6**, where there are two curved *syn*-conformeric linkers for each connecting line.

The synthesis of the metal–tbbt networks **1**–**7** is schematically presented in Scheme 1. Compounds **1**–**4** were initially synthesized with both 1:2 and 1:3 molar metal:ligand ratios. Even at the experimental M:L ratio of 1:2, the structures of **1**–**4** with an M:L formula ratio of 1:3 were obtained, as verified by identical powder X-ray diffractograms. However, at the ratio of 1:3, the quality of the single crystals for the X-ray analysis was usually better. The synthesis of **1**–**4** and **6** proceeds by the straightforward reaction of the metal salts and the tbbt linker in water. The synthesis of compound **5** includes the combination of three separately heated solutions of the metal salt, NH_4_SCN, and linker. The synthesis of **7** includes the in situ formation of an intermediary product (ferrous nitrate) [76] at elevated temperature before the addition of tbbt. The combination of hot solutions in the case of **5**–**7** was necessary to avoid the rapid precipitation of **5**–**7** as powders, instead of the formation of single crystals upon slow cooling (Scheme 1). Images of the single crystals can be found in Supplementary Materials, Section S5. The colorless, off-white to yellow-orange color of the iron products and the yellow to orange color of the cobalt compounds signals their HS state [25].

The IR spectra of compounds **1**–**7** (see Supplementary Materials, Section S3) show the characteristic bands of the linker for ν(C=C) at around 969 cm^−1^ and ν(C=N) at about 1515 cm^−1^. Also, the indicative anion bands can be observed (ν(B–F): 1045 cm^−1^ for **1** and **2**; ν(Cl–O): 1091 cm^−1^, 936 cm^−1^, 621 cm^−1^ for **3** and **4**; ν(NCS): 2048 cm^−1^ for **5** and **6**) [77]. Due to structural similarities, the IR spectra of **1**–**4** or **5** and **6**, respectively, look almost identical.

Thermogravimetric analysis indicates a thermal stability of at least 250 °C for **1**–**6** and around 80 °C for **7** (see Supplementary Materials, Section S4). The main thermal decomposition step for **1**–**7** is attributed to the decomposition of the tbbt linker. The neat tbbt compound decomposes between ~180 and 280 °C at a heating rate of 5 K min^−1^ (Figure S4). As a linker in the metal complexes **1**–**7**, the decomposition starts at ~250 °C or even later, indicating a thermal stabilization upon metal coordination caused by rigidification of the molecule. The BF_4_^–^ compounds **1** and **2** (dec. from ~300 °C, Figures S12 and S13) or the NCS compounds **5** and **6** (dec. starting ~250 °C, Figures S16 and S17) show almost similar stabilities and TGA traces. Decomposition of the BF_4_^–^ anion leads to FeF_3_ in **1**, CoF_2_ in **2**, FeS_2_ in **5**, and CoS_2_ in **6**, whose mass fractions are close to the residual mass-% values at 600 °C. Perchlorate compounds **3** and **4** both undergo sudden sharp mass loss because of an explosive decomposition at about 250 °C and 340 °C, respectively (Figures S14 and S15). Please note our warning that perchlorate salts are potentially explosive and must be handled with care! The first decomposition step in **7** is due to the loss of the four water molecules, while the second step at above 300 °C is attributed to the ligand (Figure S18).

The pH stability of each compound was visually checked in an aqueous medium for one hour. Iron compounds **1**, **3**, **5**, and **7** are stable within a range of pH 4 to 6, while the cobalt analogues **2**, **4**, and **6** are stable between pH 1 and 8. Below pH 3 and above pH 7, the crystals of the iron compounds very quickly turned from almost colorless to green. The cobalt compounds turn blue above pH 8 but retain their faint yellow-orange color down to pH 1.

The topology of the metal-linker networks was elucidated by single-crystal X-ray analysis as presented below. Additional crystal data and values of the distortion of the metal coordination polyhedra of **1**–**7** can be found in the Supplementary Materials, Sections S6 and S7. Additional structure images are provided in the Supplementary Materials, Section S8. For each compound powder, X-ray diffraction patterns positively matched the corresponding simulated pattern from the single-crystal X-ray analysis and, thereby, supported the phase purity of each compound (see Supplementary Materials, Section S9).

### 3.1. Crystal Structures of [M(tbbt)_3_](BF_4_)_2_ and [M(tbbt)_3_](ClO_4_)_2_ (M = Fe **1**, **3**; M = Co **2**, **4**)

**Figure 1:**
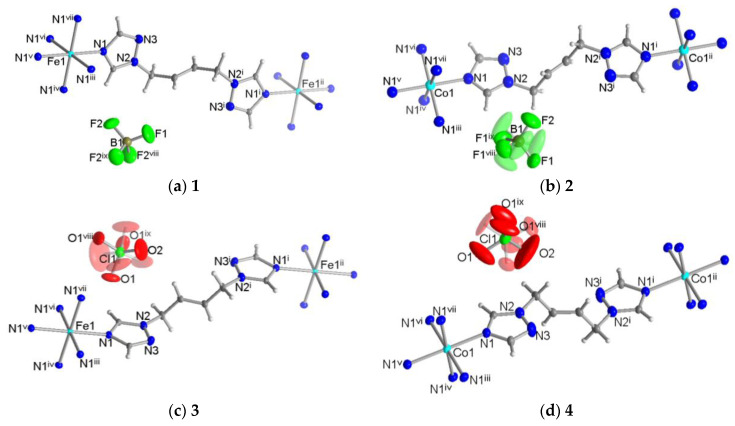
Extended asymmetric unit of (**a**) **1**, (**b**) **2**, (**c**) **3,** and (**d**) **4** (50% thermal ellipsoids and H atoms with arbitrary radii). The anion disorder is indicated by transparent atoms. The symmetry transformations are as follows: (**a**) i = 1 − x, 2 − y, 2 − z; ii = 1 + x, y, 1 + z; iii = −1 − x + y, 1 − x, z; iv = −1 + y, −x + y, 1 − z; v = −x, 2 − y, 1 − z; vi = 1 + x − y, 1 + x, 1 − z; vii = 1 − y, 2 + x − y, z; viii = −x + y, 1 − x, z; ix = 1 − y, 1 + x − y, z. (**b**) i = 2 − x, 1 − y, 2 − z; ii = x, −1 + y, 1 + z; iii = 1−x + y, 1 − x, z; iv = 1 − y, x − y, z; v = 2 − x, 2 − y, 1 − z; vi = 2 − y, 1 + x − y, z; vii = 1 − x + y, 2 − x, z; viii = 1 − x + y, 1 − x, z; ix = 1 − y, x − y, z. (**c**) i = 1 − x, 1 − y, −z; ii = −1 + x, −1 + y, −1 + z; iii = 2 − y, 1 + x − y, z; iv = 1 + x − y, x, 1 − z; v = 2 − x, 2 − y, 1 − z; vi = y, 1 − x + y, 1 − z; vii = 1 − x + y, 2 − x, z; viii = 1 − y, 1 + x, z; ix = −x + y, 1 − x, z. (**d**) i = 1 − x, −y, 2 − z; ii =1 + x, y, 1 + z; iii = −y, x − y, z; iv = −x + y, −x, z; v = −x, −y, 1 − z; vii = x − y, x, 1 − z; vi = y, −x + y, 1 − z; vii = 1 − x + y, 1 − x, z; ix = 1 − y, x − y, z.

Crystallographic X-ray analysis shows compounds **1**–**4** to be isostructural, all crystallizing in the trigonal space group *P*$\bar{3}$. The asymmetric unit contains one M(II) atom (M = Fe, Co) (residing on an inversion center and a threefold rotation axis), one half-linker molecule (inversion center at the middle of the C=C bond) and half a BF_4_^–^ or ClO_4_^–^ anion on the threefold rotation axis with one of the B–F or Cl–O bonds coinciding with the rotation axis (Figure 1). The F and O atoms of the anion in **2**–**4** are slightly disordered, as indicated by the transparent F or O atoms in Figure 1. The octahedral coordination sphere of each Fe(II) and Co(II) metal ion is composed of six symmetry-related nitrogen donor atoms from six triazole rings of six tbbt molecules (Figure 1) and is only negligibly distorted (see Supplementary Materials, Section S7).

The Fe–N bond lengths are 2.186(2) Å in **1** and 2.191(2) Å in **3**, which is the typical range of an iron(II) high-spin complex [26,75,78]. The Co–N bond lengths with 2.149(2) Å in **2** and 2.150(2) Å in **4** are also in the range of a cobalt(II) high-spin complex [78,79].

**Figure 2:**
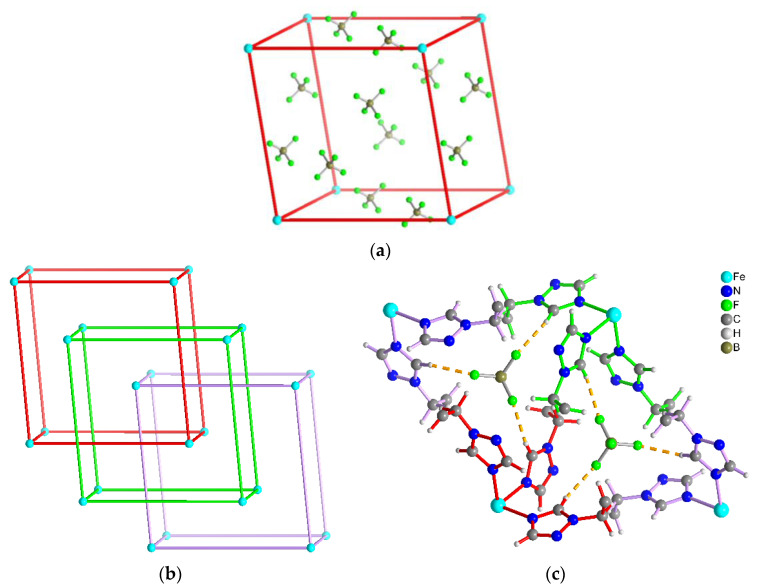
(**a**) Schematic presentation of the primitive lattice metal-linker topology (**pcu**) in **1** (identical to **2**–**4**) with the positions of the anions on the faces of the primitive lattice. (**b**) Threefold interpenetration of three symmetry-related primitive lattices, differentiated by color (the anions are not shown for clarity). (**c**) Interpenetration-guiding template effect of the BF_4_^–^ (and similarly of the ClO_4_^–^) anions through (triazole)C–H⋯F hydrogen bonds (orange dashed lines) to triazole moieties from three different networks of **1** (for a presentation of the (triazole)C–H⋯O hydrogen bonds in the structure of **3**, see Figure S26, Supplementary Materials).

Two crystallographically equivalent metal(II) atoms are connected by one ligand molecule to establish a three-dimensional (3D) network, which forms a primitive, almost cubic lattice (**pcu**) with two non-coordinated anions on each face (Figure 2a). The M⋯M distance along the connecting tbbt linker is between 13.9 Å and 14.0 Å. In view of the long linker and, consequently, large void space in a single 3D network, three such symmetry-related 3D networks interpenetrate (Figure 2b) [80,81,82,83,84,85], as was described in the literature for bis(1,2,4-triazole)- and bis(imidazole)metal compounds [58,86]. The BF_4_^–^ or ClO_4_^–^ anions in **1**–**4** control or guide the interpenetration of the 3D frameworks because each anion forms three (triazole)C–H⋯F (2.27 Å–2.48 Å), respectively, (triazole)C–H⋯O hydrogen bonds (2.37 Å–2.65 Å) with three tbbt ligands, each belonging to one of the three interpenetrating networks (Figure 2c). The BF_4_^–^ or ClO_4_^–^ anions give rise to the same structural motif in **1**–**4** because of their similar size, shape, and hydrogen-bonding capability. Consequently, different structures would be expected with other charge-compensating counter anions [87,88]. There are no voids in the structure. A calculation of voids with the Mercury program [66] yielded only about 11–13 Å^3^ or 1.2–1.4% of empty space per unit cell volume with a probe radius of 1.2 Å in **1**–**4**.

### 3.2. Crystal Structures of [M(NCS)_2_(tbbt)_2_] (M = Fe **5**; M = Co **6**)

**Figure 3:**
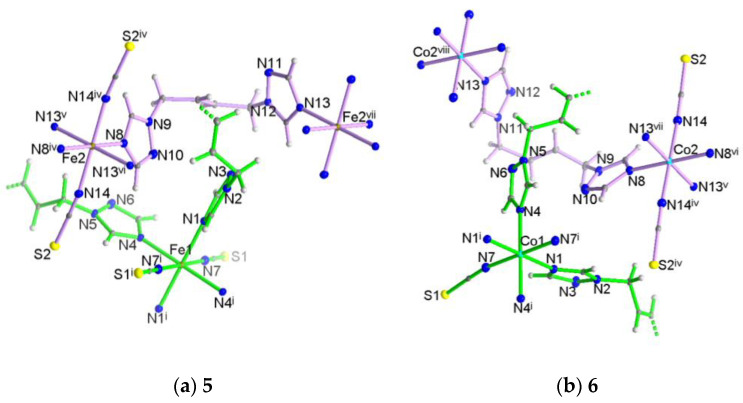
Extended asymmetric unit of (**a**) **5** and (**b**) **6**. Layers are displayed in green and chains in purple color (50% thermal ellipsoids and H atoms with arbitrary radii). Symmetry transformations in (**a**): i = 1 − x, 1 − y, 1 − z; ii = 1 − x, 1 − y, −z; iii = 2 − x, −y, 1 − z; iv = 1 − x, −y, −z; v = x, −1 + y, z; vi = 1 − x, 1 − y, −z; vii = x, 1 + y, z; in (**b**): i = 1 − x, 1 − y, −z; ii = −x, 2 − y, −z; iii = 1 − x, 1 − y, 1 − z; iv = 1 − x, 2 − y, 1 − z; v = x, 1 + y, z; vi = 1 − x, 2 − y, 1 − z; vii = 1 − x, 1 − y, 1 − z; viii = x, −1 + y, z.

The isostructural compounds **5** and **6** crystallize in the triclinic space group *P*$\bar{1}$. The asymmetric unit contains two crystallographically different M(II) atoms (M = Fe, Co), each on an inversion center, one full and two half tbbt molecules (the latter have again an inversion center at the middle of the C=C bond), and a metal-coordinated isothiocyanate anion (Figure 3). Both metal atoms are coordinated in an octahedral fashion by four different tbbt molecules and two *trans*-positioned isothiocyanate anions [75,78]. Different from the structures of **1**–**4** where the anions BF_4_^−^ and ClO_4_^−^ are weakly coordinating and normally do not coordinate to metal atoms, the well-ligating NCS^–^ anions coordinate through the N atom as isothiocyanate ligands to the metal atoms. Thus, for the six-coordinated metal atoms, only four donor atoms have to come from the tbbt molecules so that a 3D metal-linker network will not be feasible anymore—at most, only a 2D network. The coordination sphere for the M2 atom is slightly more distorted than for M1 (see Supplementary Materials, Section S7).

The bond lengths in **5** are Fe–NCS 2.139(1) Å and Fe–N(triazole) 2.201(1) Å, and therefore, both are in the typical HS range. The Fe–NCS bond length is the shorter one in accordance with the literature [26,75,78]. In **6**, the Co–NCS bond length is 2.110(2) Å and Co–N(triazole) 2.158(2) Å, both also indicating the HS state [78,79].

**Figure 4:**
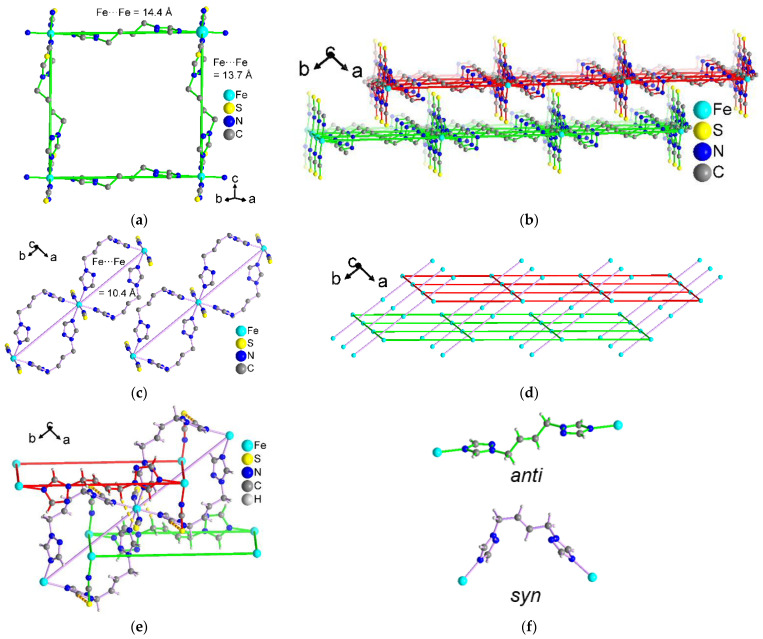
Packing arrangement in **5** (M = Fe) (identical in **6**, M = Co; hydrogen atoms omitted for clarity). Section of the **sql** layer substructure as (**a**) on-top view and (**b**) side view with two layers differentiated by red and green colors. (**c**) Two parallel chains. (**d**) Schematic arrangement of layers and chain (NCS ligands omitted). (**e**) Polycatenation organized by (triazole)C–H⋯S and (butene)C–H⋯S hydrogen bonds as orange and golden dashed lines, respectively. (**f**) *Anti* and *syn* conformation of the tbbt linker in the layers and in the chains, respectively.

The two half-linker molecules connect the M1 atoms to form a two-dimensional **sql**-lattice. The M⋯M distances within the layers for both compounds **5** and **6** lie between 13.6 Å and 14.4 Å (Figure 4a). These layers are arranged in an AB stacking sequence with a distance of 6.4 Å between the layer planes, as defined by the Fe atoms (Figure 4b). The NCS ligands point up and down each layer. The crystallographically complete tbbt linkers connect the M2 atoms to linear 1D chains with M⋯M distances of 10.4 Å (M = Fe) and 10.3 Å (M = Co) (Figure 4c). These 1D chains polycatenate the 2D layers to a 3D framework (Figure 4d) [80,81,82,83,84,85]. The coordinated isothiocyanate anions organize this polycatenation by the formation of (triazole)C–H⋯S and (butene)C–H⋯S hydrogen bonds (C⋯S = 2.70 Å–3.01 Å) from the C–H donors in the chains to the NCS ligands in the 2D layers (Figure 4e). Such chains, which interact with layers over C–H⋯S H-bonds, can be found as a structure motif in many different triazole-based coordination polymers [48,58,59,60,61,89,90]. The conformation of tbbt is *syn* for the chains and *anti* in the layers of structures **5** and **6** (Figure 4f) [89]. The same mixture of 2D networks and 1D chains is found in [Fe(NCS)_2_(btb)_2_] (btb = 1,4-bis(1,2,4-triazol-1-yl)butane) [60].

### 3.3. Crystal Structure of [Fe(H_2_O)_2_(tbbt)_2_]Br_2_·2H_2_O (**7**)

**Figure 5:**
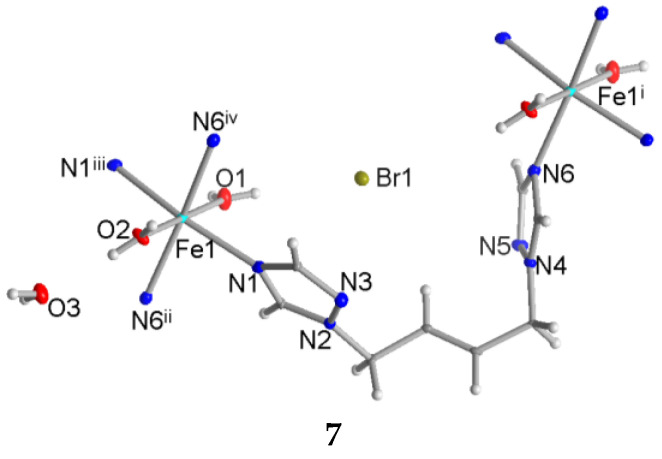
Extended asymmetric unit of **7** (50% thermal ellipsoids and H atoms with arbitrary radii). Symmetry transformations: i = x, 1/2 + y, 1/2 + z; ii = x, −1/2 + y, −1/2 + z; iii = 1 − x, 1 − y, z; iv = 1 − x, 3/2−y, −1/2 + z.

Using bromide as the counter anion, a different structure motif was obtained. Compound **7** crystallizes in the orthorhombic space group *Fdd2*. The asymmetric unit contains one-half of an octahedrally coordinated iron(II) atom (on a twofold rotation axis), one full linker molecule, two halves of Fe-coordinated water molecules (aqua ligands), one non-coordinated crystal water molecule, and a bromine anion (Figure 5). In contrast to compounds **1**–**4** and similar to compounds **5** and **6**, the metal ion in compound **7** is only coordinated by four nitrogen donor atoms. Hence, a 3D framework cannot form, only a 2D metal-linker network. The potentially coordinating Br^–^ anion does not bind to iron and engages only in hydrogen bonding. Possibly, the coordination of the harder aqua ligand and the non-coordination of the softer bromine can be rationalized by the hard and soft acid and base concept if the Fe(II) ion is categorized on the harder acid site [91,92,93]. The distortion of the octahedral coordination environment is stronger compared to compounds **1**–**6** (see Supplementary Materials, Section S7).

The Fe–N1 bond length is 2.18(3) Å; the Fe–N6 bond length is slightly larger with 2.20(3) Å. Both bond lengths signal the high-spin state of Fe(II) in this compound [26,75,78]. The bond length between Fe and O2 with 2.098(2) Å is slightly shorter than the Fe–O1 bond length with 2.140(5) Å.

**Figure 6:**
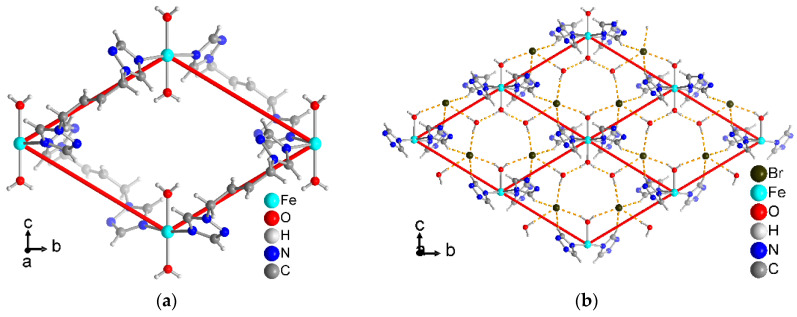

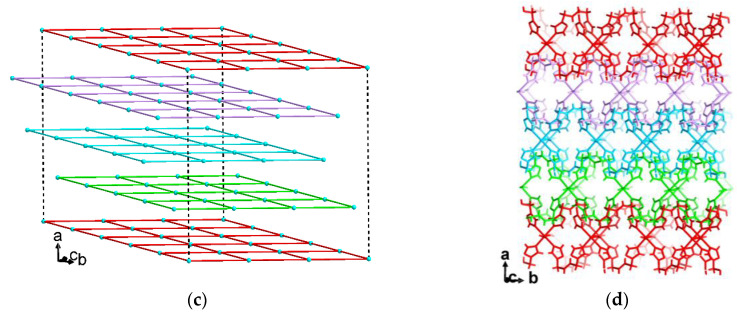
Illustration of a section of the **sql** layer in the structure of **7** with (**a**) a single opening depicting the *syn*-conformation of the tbbt linker and (**b**) showing the filling with crystal water molecules and bromide ions (hydrogen bonds as dashed orange lines; from the linker only the triazole part is shown for clarity). (**c**) ABCD stacking of the **sql** layers as represented by Fe atoms and their topological connections (the black dotted lines are guides to the eye). (**d**) ABCD stacking of the **sql** layers showing their interdigitation.

In **7**, corrugated **sql** layers are formed with a *syn* conformation of the linker (Figure 6a), whereas in **1**–**6**, the tbbt linker was bridging the metal atoms in the networks with its *anti*-conformation (only the chains in **5** and **6** had the linker bridge in *syn*-conformation). In **7**, the *syn* conformation is responsible for the layer corrugation. Hence, in **7**, the Fe⋯Fe distance of 9.6 Å within the layer is considerably smaller than the approximately 14 Å in the 3D networks in **1**–**4** and in the layers in **5**–**6** but is as seen in the chains in **5**–**6**. Consequently, no interpenetration or polycatenation occurs with the layers in **7**; instead, each pore in the layer is filled with four water molecules and two bromine anions (Figure 6b). These corrugated layers are arranged in an ABCD stacking type with a distance of 7.7 Å between the layer planes as defined by the Fe atoms (Figure 6c). This distance is almost 1.4 Å larger than in **5** and **6**. The different layers interdigitate because of their corrugated nature (Figure 6d). The hydrogen bond length between the coordinated and non-coordinated water molecules is 1.94(5) Å. The O–H⋯Br hydrogen bonds are between 2.48(4) Å and 2.61(4) Å. The (triazole)C–H⋯Br hydrogen bonds are 2.83(5) Å and 2.86(5) Å.

### 3.4. Spin-Crossover Properties

The LS state is enthalpically favored, hence more stable at lower temperatures, while the HS state is entropically favored and, therefore, more stable at higher temperatures [26]. Given that Fe(II) triazole complexes are known to be thermochromic, with a reddish-purple color in the LS state, compounds **1**–**7** were cooled from room temperature down to 77 K by immersing the vials containing off-white to yellow-orange samples into liquid nitrogen, but no color change was observed.

Considering the possibility of the occurrence of a so-called thermally induced excited spin-state trapping effect (TIESST effect), which describes a metastable HS state at low or even cryogenic temperatures on fast cooling [28,30,40,94,95], we even used a relatively low cooling rate (0.5 K/min) down to 80 K using the cryo-cooler device on the single-crystal X-ray diffractometer, but no SCO was observed.

One explanation for a non-existing SCO in **1**–**7** could be that the SCO occurs at lower temperatures or is blocked by the strong rigidity of the networks, which prevent the significant shrinking of the iron(II) nitrogen bond lengths in the LS state. Another reason for the missing SCO could be the present distortion of the metal coordination sphere (see Supplementary Materials, Section S7), which can play a significant role in the stabilization of the LS state [96,97,98,99].

**Figure 7:**
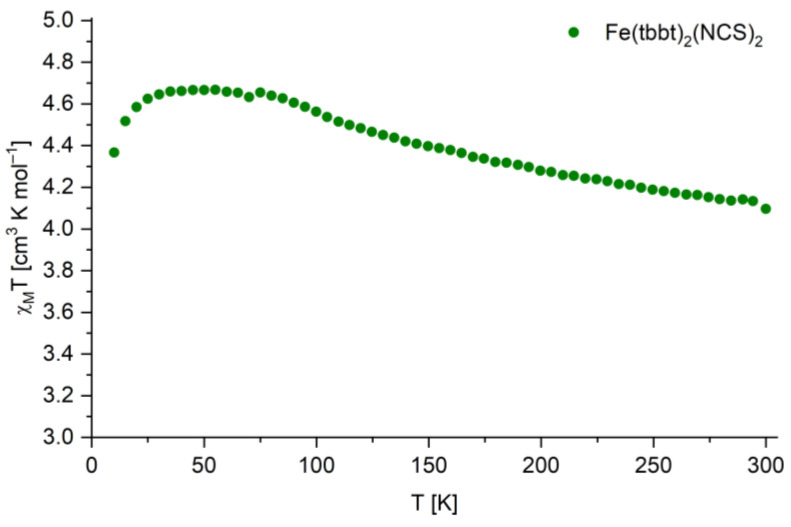
Temperature-variable magnetic susceptibility of compound **5**.

To test these assumptions, compound **5**, potentially the most structurally flexible of the iron compounds, was cooled even further to 10 K while performing SQUID measurements of the magnetic susceptibility (Figure 7).

However, even cooling to 10 K did not result in spin switching, and the compound remained in the high-spin state over the entire temperature range, suggesting that the network is still too rigid to allow SCO. A slight increase in the χ_M_*T* value is observed upon cooling to about 50 K, indicating a weak ferromagnetic interaction. The decrease with further temperature decrease can be explained by weak intermolecular antiferromagnetic interactions and/or zero-field-splitting and/or saturation of the magnetization.

Since pressure can increase the ligand field strength at the metal center by shortening the metal–ligand bond lengths, provided there is no change in the coordination polyhedron, it is well known that pressure can induce spin-crossover from the HS to the LS state [25,40,100,101]. Such a pressure-induced SCO remains less investigated because of the more challenging experimental requirements. We have thus examined the spin state of **1**–**7** under pressure [100,101]. Indeed, we were able to observe a color change from colorless to purple for compound **5**. While single crystals of **5** switched back to colorless after removing the pressure, a powder of **5** kept the purple color (Figures S34 and S35). SQUID measurements of this purple powder do not indicate any spin transition (Figure S36). A possible explanation for this color change could be a partial oxidation of iron(II) to iron (III) and/or valence tautomerism.

## 4. Conclusions

In summary, seven new iron or cobalt coordination polymers based on a bis-1,2,4-triazole ligand 1,1′-(*trans*-2-butene-1,4-diyl)bis-1,2,4-triazole (tbbt) have been successfully synthesized and structurally characterized.

The use of BF_4_^−^ or ClO_4_^−^ metal salts with weakly coordinating anions leads to the formation of triply interpenetrated 3D **pcu** lattices (**1**–**4**). As the BF_4_^−^ or ClO_4_^−^ anions do not coordinate to the metal atoms, their close-to-octahedral coordination sphere consists of nitrogen donor atoms from six different bridging tbbt linkers, thereby providing 3D frameworks. Structure and interpenetration are not controlled by interactions between the triazole rings or double bonds, as expected, but by (triazole)C-H⋯F_4_B and CH⋯O_4_Cl hydrogen bonds through a template effect of the non-coordinated BF_4_^–^ or ClO_4_^–^ anions. In compounds **5** and **6**, the used SCN^–^ is coordinated directly to the metal center, with only four N atoms from four tbbt linkers coordinating to the metal atoms. Consequently, these two structures are built of 2D **sql** layers and 1D double-bridged chains. The 2D layers are interpenetrated by the 1D chains, with the interpenetration again controlled and stabilized by hydrogen bonds from the linkers of the chains to the isothiocyanate groups in the layers. Thus, both in **1**–**4** and in **5** and **6**, the anions exert an interpenetration-guiding template effect. When Br^–^ is used as a counter ion, like in **7**, non-interpenetrated **sql** layers in an ABCD stacking type are obtained. In contrast to **1**–**6**, the metal center is coordinated by four nitrogen atoms of linker molecules and two water molecules, instead of six nitrogen atoms. The bromine anion does not coordinate to the iron atoms. Instead, the presence of hydrogen-bonded Br^−^ and water molecules in the openings of the **sql** layers prevent any interpenetration in **7**.

From the existing interpenetration-guiding template effects and various structural motifs, it was evident in this work that the coordinated and non-coordinated anions play an essential role in the formation of the corresponding networks.

SCO is not observable in any compound. The color change of **5** to purple under pressure seems not to correlate with any spin transition. To understand this behavior, further experiments will follow in subsequent research.

## Data Availability

The data presented in this study are available on request from the corresponding author. The CCDC numbers 2280632–2280638 for **1**–**7** contain the supplementary crystallographic data reported in this paper. These data can be obtained free of charge from the Cambridge Crystallographic Data Centre via www.ccdc.cam.ac.uk/data_request/cif (accessed on 30 July 2023).

## Supplementary Materials

The following supporting information can be downloaded at https://www.mdpi.com/article/10.3390/polym15153286/s1: Section S1: Used Chemicals; Section S2: Ligand analyses; Section S3: Infrared spectra of **1**–**7**; Section S4: Thermogravimetric analyses of **1**–**7**; Section S5: Crystal images of **1**–**7**; Section S6: Crystal data of **1**–**7**; Section S7: Distortion of the coordination polyhedra of **1**–**7;** Section S8: Additional structure images; Section S9: Powder X-ray diffraction patterns of **1**–**7**; Section S10: Pressure experiments of **5**; References [72,96,97,98,99,102,103,104,105] are cited in the Supplementary Materials.
