# Supplementary material for: Interpenetration Phenomena via Anion Template Effects in Fe(II) and Co(II) Coordination Networks with a Bis-(1,2,4-triazole) Ligand
Source: Polymers (Basel). 2023 Aug 3;15(15):3286. doi: 10.3390/polym15153286 (PMC10422438; doi:10.3390/polym15153286)
Supplement: Supplementary file 1 [file polymers-15-03286-s001.zip › polymers-2525699-supplementary.pdf]

Supplementary Materials

S1. Used chemicals

Table S1. Overview of used chemicals.

Chemical	Supplier	Purity
1,2,4-triazole	BLDpharm	97%
Acetonitrile	Riedel-de Haën	99.9%
Ascorbic acid	Roth	99%
$Ba(NO_3)_2$	lab inventory	–
Buffer solutions (pH 4 to 8)	AppliChem	–
Chloroform	Fisher	99.8%
$Co(BF_4)_2{\cdot}6H_2O$	Alfa Aesar	96%
$Co(ClO_4)_2{\cdot}6H_2O$	Strem Chemicals Inc.	–
Co(SCN)	Johnson Matthey Alfa Products	98%
DSMO-d^6	Sigma-Aldrich	99.9%
Ethanol	Chemsolute	99.9%
$Fe(BF_4)_2{\cdot}6H_2O$	abcr	97%
$Fe(ClO_4)_2{\cdot}xH_2O$	Sigma-Aldrich	98%
$FeSO_4{\cdot}2H_2O$	Merck	p.a.
KBr	Riedel-de Haën	99.5%
KOH	Merck	p.a.
$MgSO_4$	VWR Chemicals	98.0%
$(NH_4)_2Fe(SO_4)_2{\cdot}6H_2O$	Sigma-Aldrich	99%
NH_4SCN	Riedel-de Haën	99%
trans-1,4-dibromo-2-butene	BLDpharm	98%

S2 . Ligand analyses

Before the analyses the sample was dried in vacuo at 60 °C as otherwise water signals will be detected.

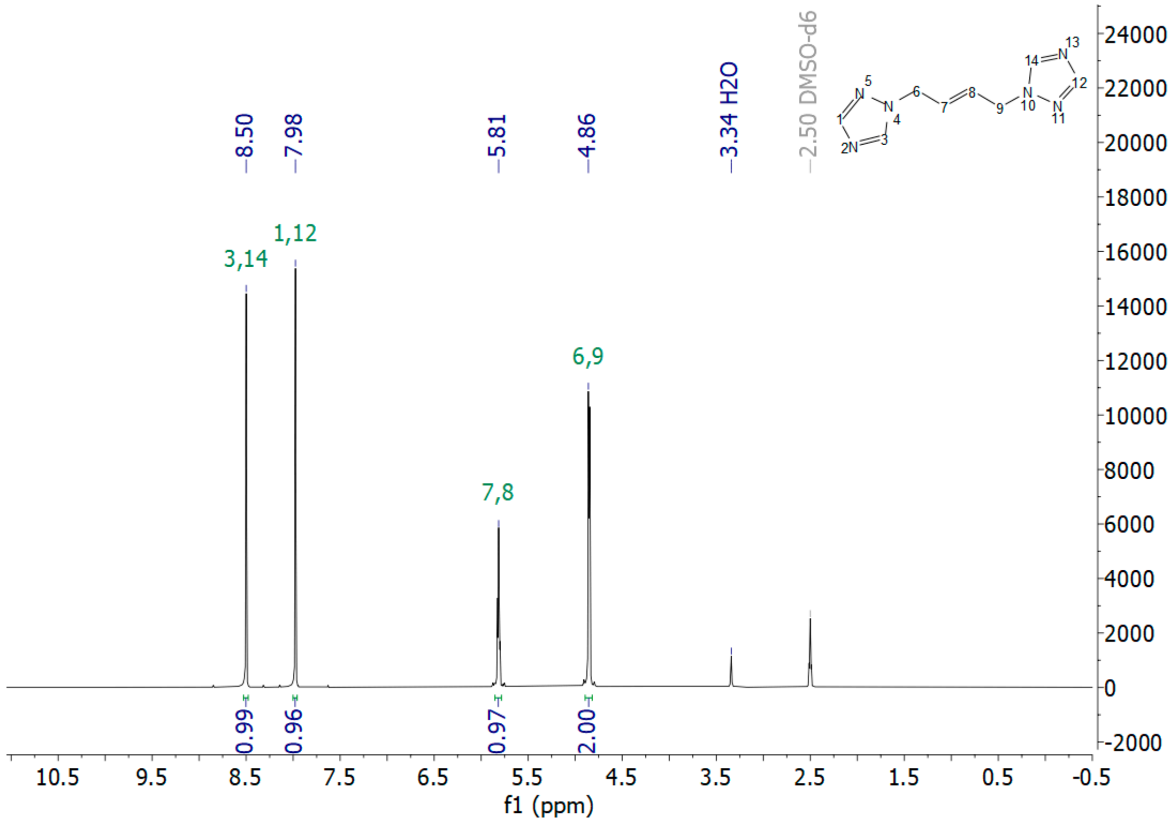

Figure S1. ^{1}H-NMR spectrum (300 MHz, DMSO-d_6) of tbbt. The water peak is due to the difficult-to-avoid water content of the used DMSO-d_6.

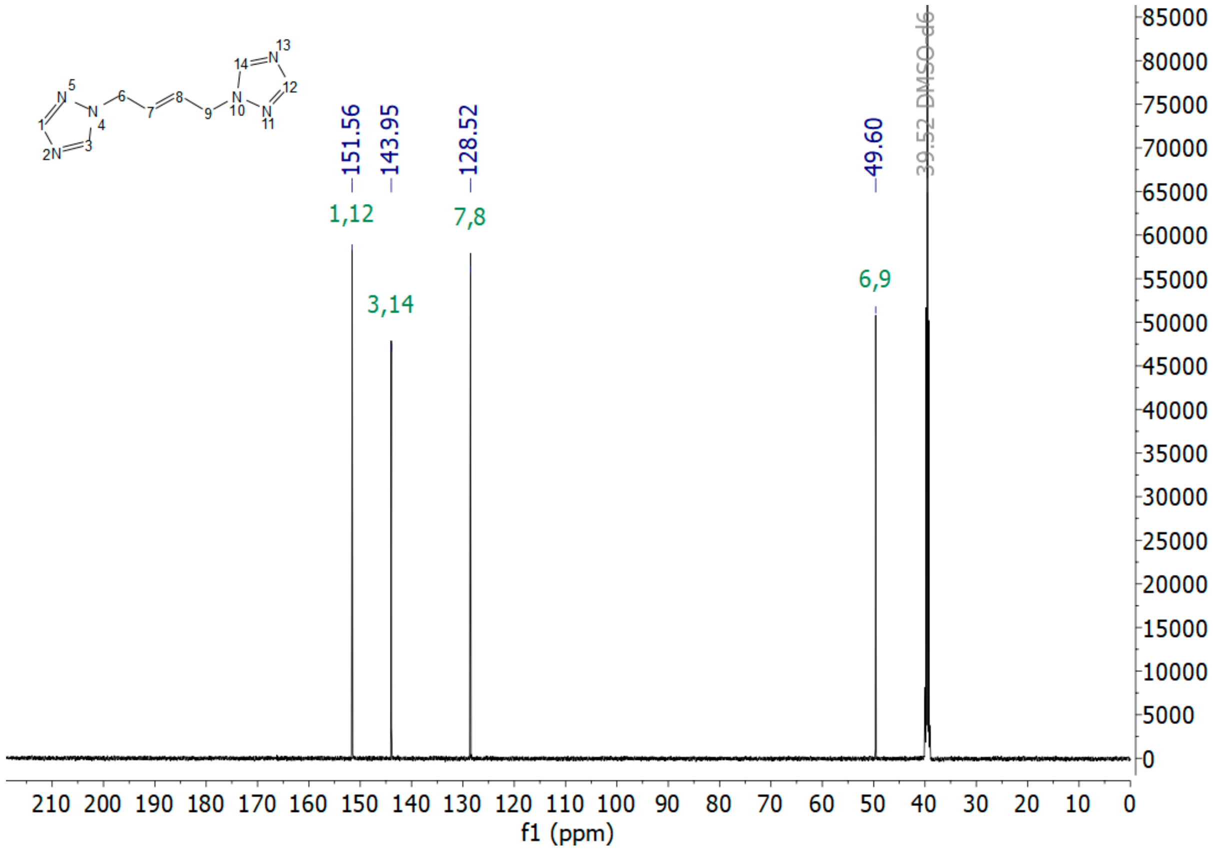

Figure S2. $^{13}C\{^{1}H\}$-NMR spectrum (75 MHz, DMSO-d_6) of tbbt.

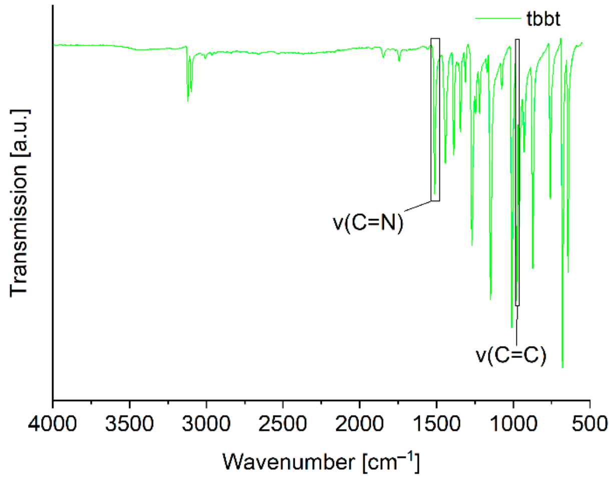

Figure S3. IR spectrum of tbbt (ATR).

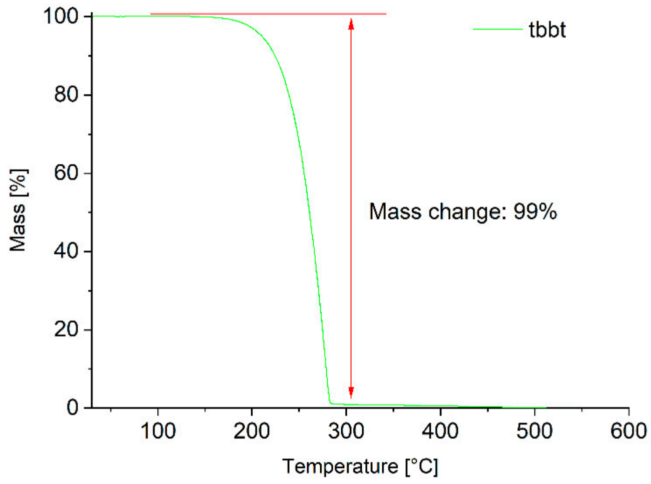

Figure S4. TGA of tbbt (heating rate 5 K min^{-1}). Before the analysis the sample was dried in vacuo at 60 °C as otherwise a mass loss of up to 8% below 100 °C will be detected due to absorbed water.

S3. Infrared spectra of 1-7

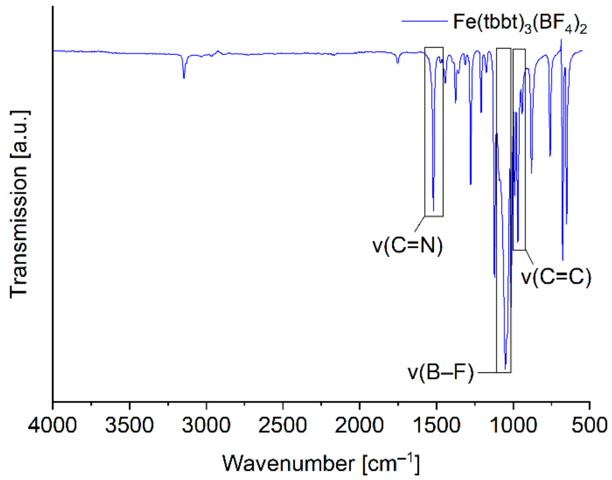

Figure S5. IR spectrum of **1** (ATR).

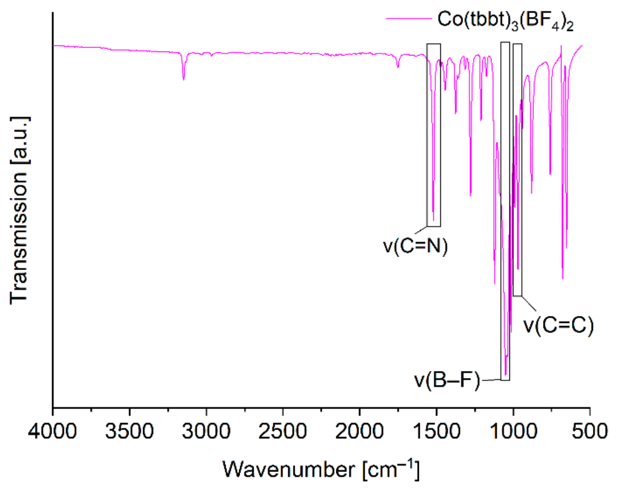

Figure S6. IR spectrum of **2** (ATR).

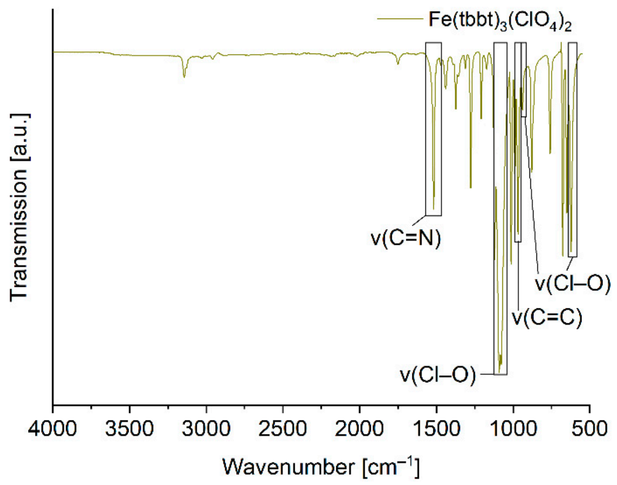

Figure S7. IR spectrum of **3** (ATR).

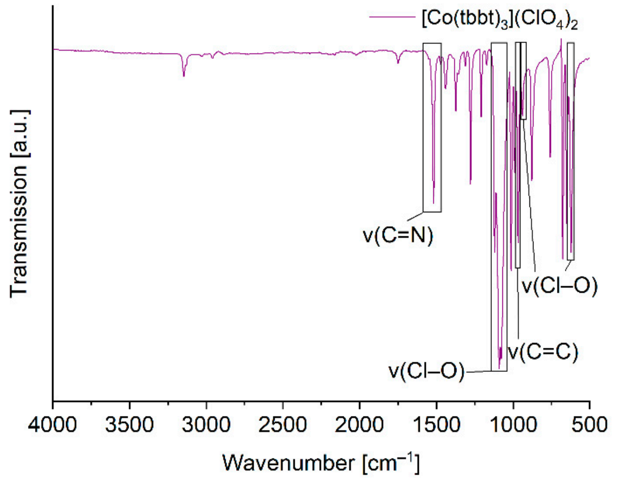

Figure S8. IR spectrum of **4** (ATR).

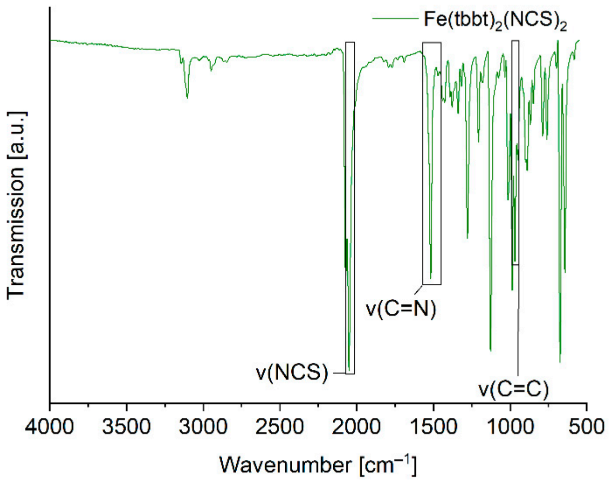

Figure S9. IR spectrum of **5** (ATR).

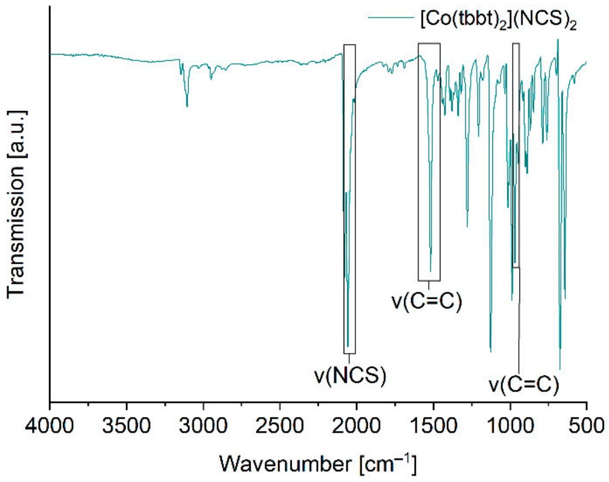

Figure S10. IR spectrum of **6** (ATR).

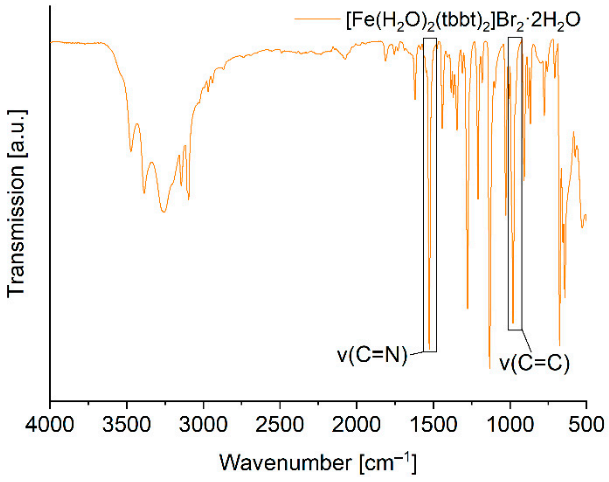

Figure S11. IR spectrum of **7** (ATR).

S4. Thermogravimetric analyses of 1-7

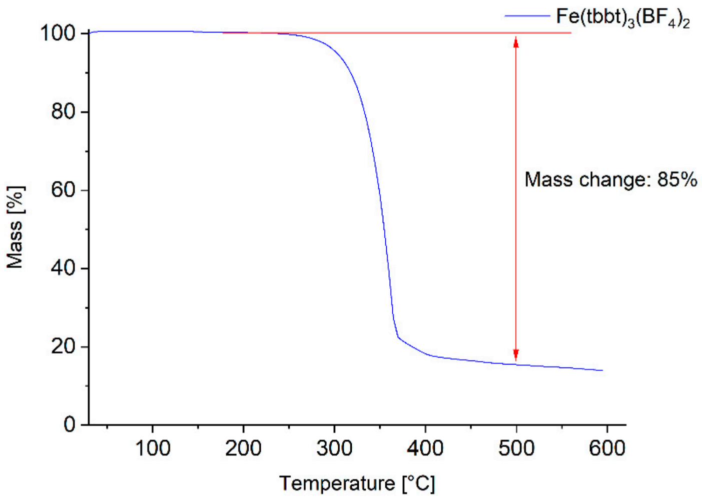

Figure S12. TGA of **1** (heating rate 5 K min^{-1}). The theoretical mass loss of three tbbt ligands would be 71%, leaving a residual mass for $Fe(BF_4)_2$ of 29%. Decomposition of BF_4 would leave a residual mass for FeF_2 of 12% and for FeF_3 of 14%.

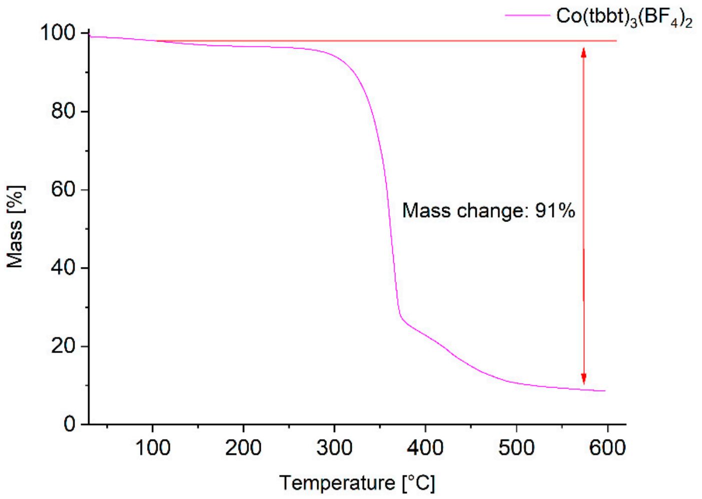

Figure S13. TGA of **2** (heating rate 5 K min^{-1}). The theoretical mass loss of three tbbt ligands would be 71%, leaving a residual mass for $Co(BF_4)_2$ of 29%. Decomposition of BF_4 would leave a residual mass for CoF_2 of 12%.

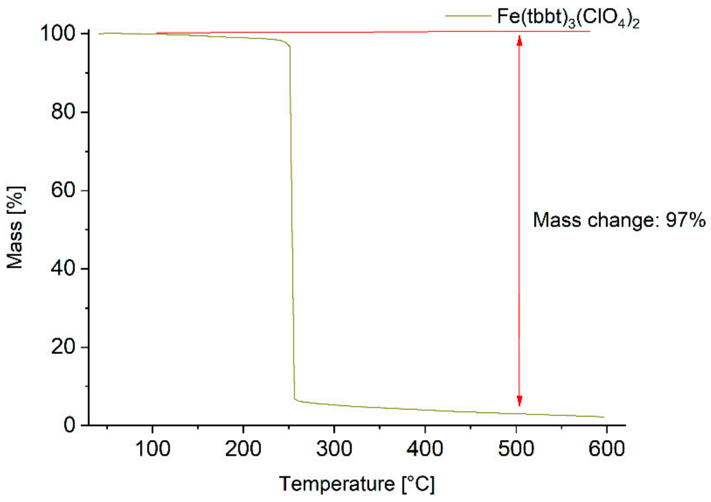

Figure S14. TGA of **3** (heating rate 5 K min^{-1}). The sudden sharp mass loss at about 250 °C with essentially no residual mass is due to an explosive decomposition which blew the sample out of the sample holder.

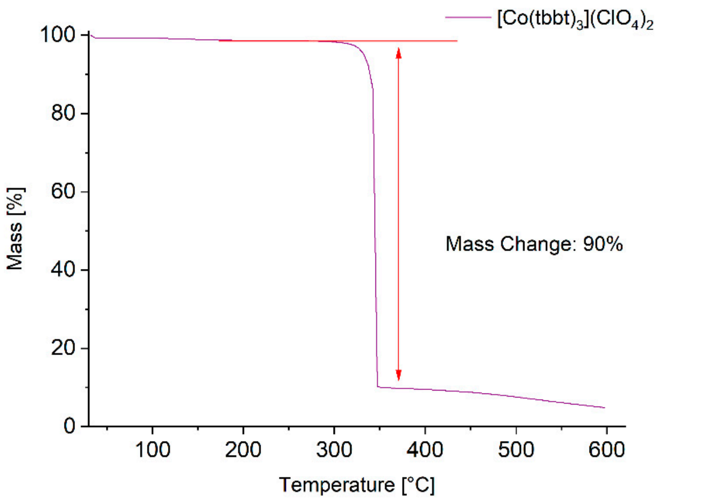

Figure S15. TGA of **4** (heating rate 5 K min^{-1}). The sudden sharp mass loss at about 340 °C with essentially no residual mass at 600 °C is due to an explosive decomposition which blew the sample out of the sample holder.

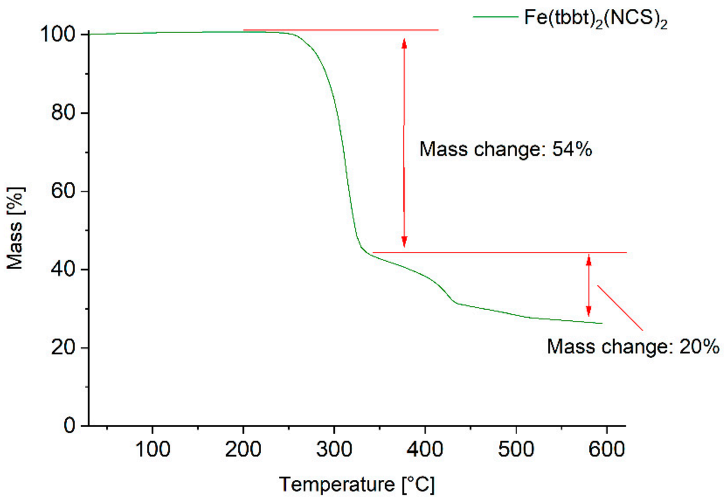

Figure S16. TGA of **5** (heating rate 5 K min⁻¹). The theoretical mass loss of two tbbt ligands would be 69%, leaving a residual mass for $Fe(NCS)_2$ of 31%. Decomposition of NCS would leave a residual mass for FeS or 16% or for FeS_2 of 22%.

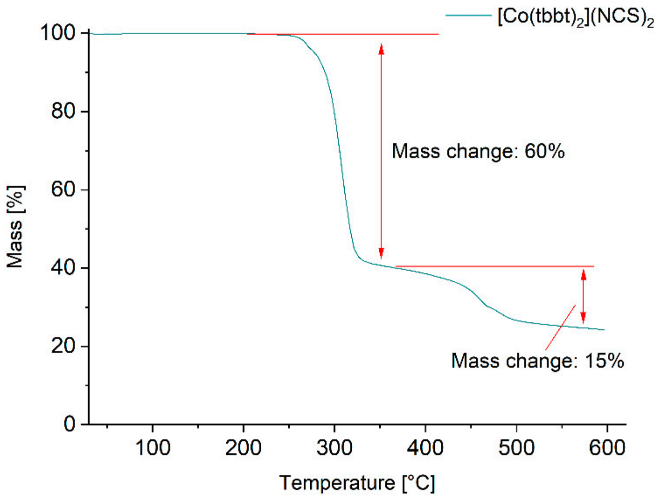

Figure S17. TGA of **6** (heating rate 5 K min^{-1}). The theoretical mass loss of two tbbt ligands would be 68%, leaving a residual mass for $Co(NCS)_2$ of 32%. Decomposition of NCS would leave a residual mass for CoS or 16% or for CoS_2 of 22%.

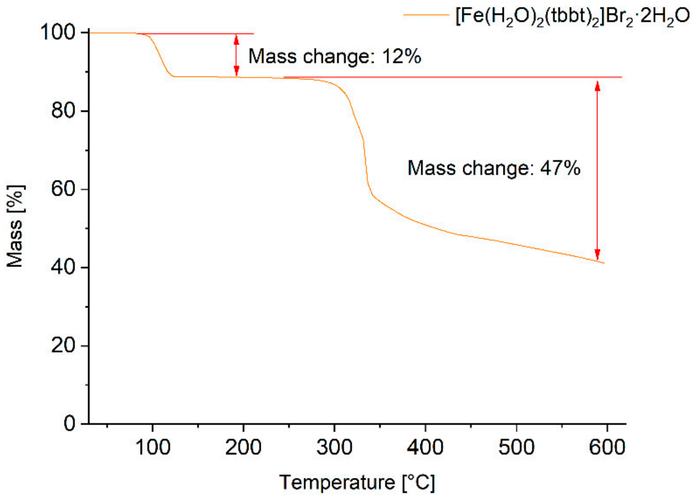

Figure S18. TGA of **7** (heating rate 5 K min^{-1}). The calculated mass loss of $4H_2O$ molecules per formula unit is 11% (obs. 12%). The calculated residual mass for $FeBr_2$ would be 32%. We note that the mass loss is not complete and still continuing with a strong negative slope at 600 °C.

S5. Crystal images

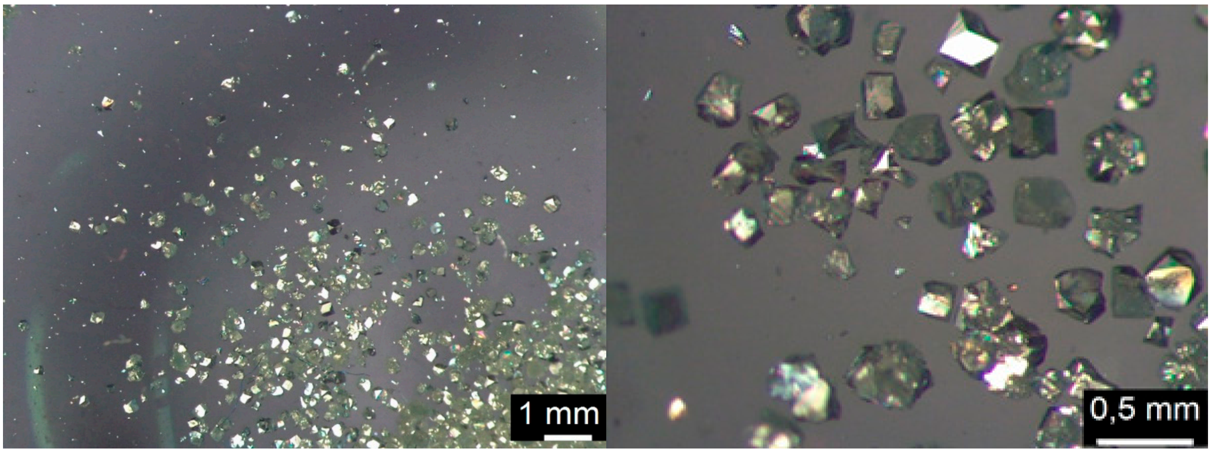

Figure S19. Crystal images of **1**.

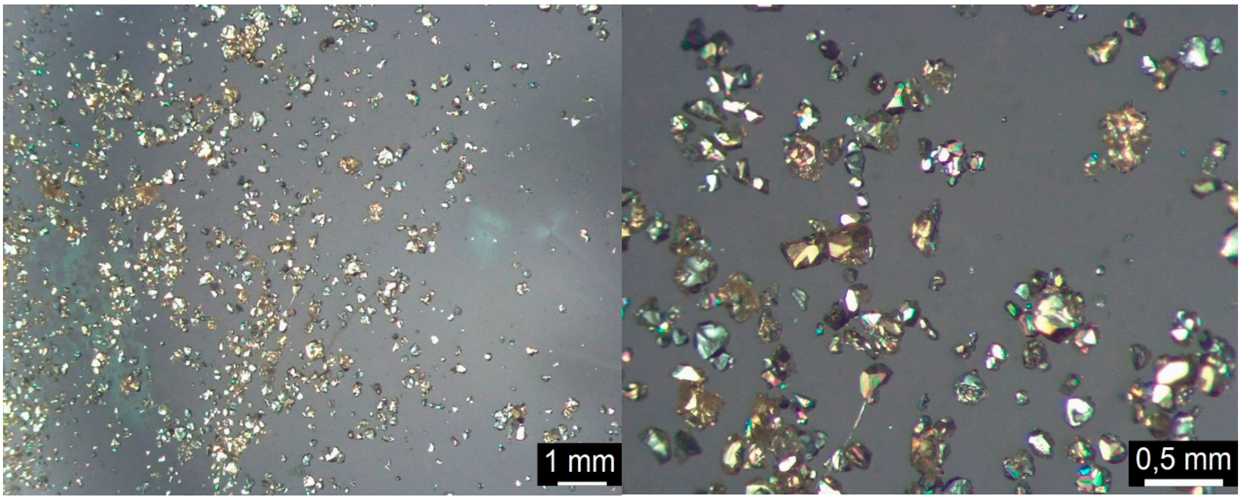

Figure S20. Crystal images of **2**.

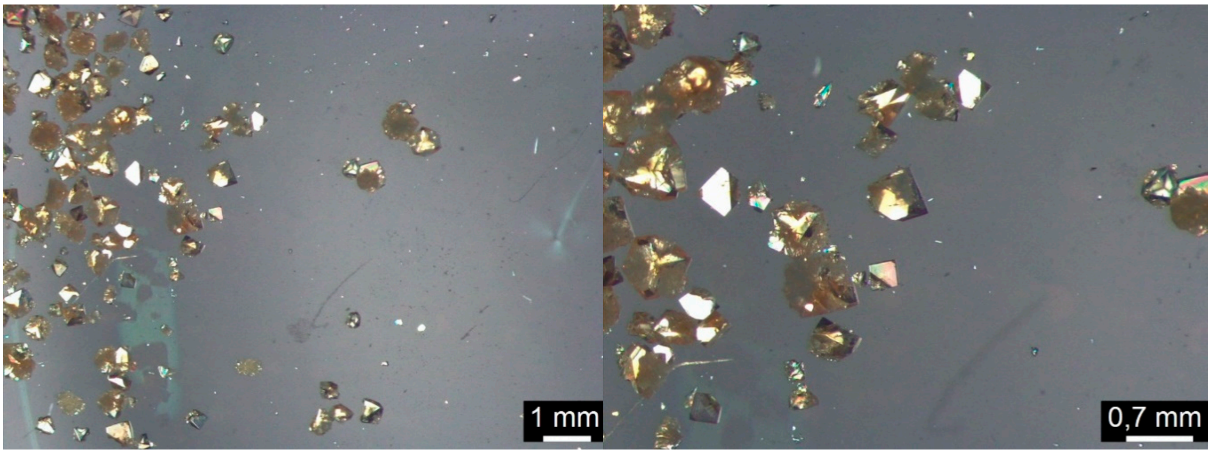

Figure S21. Crystal images of **3**.

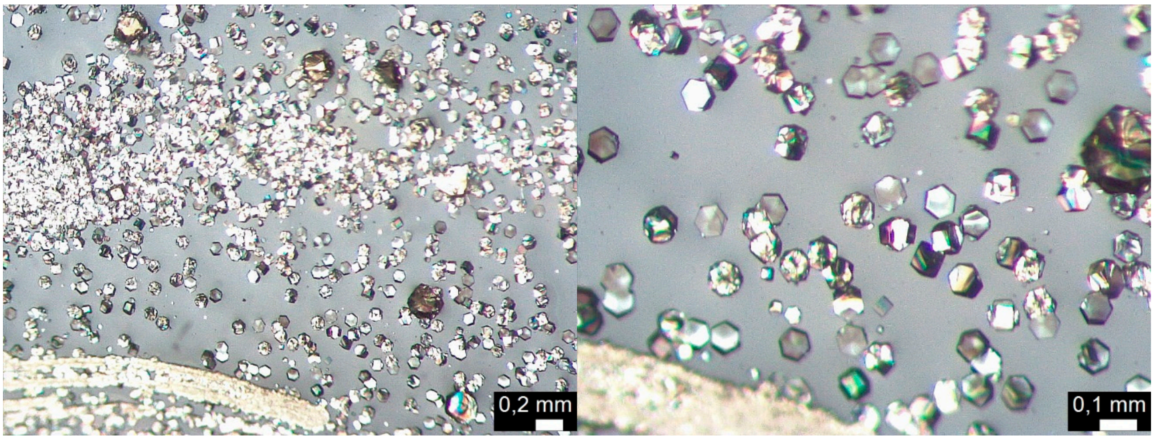

Figure S22. Crystal images of **4**.

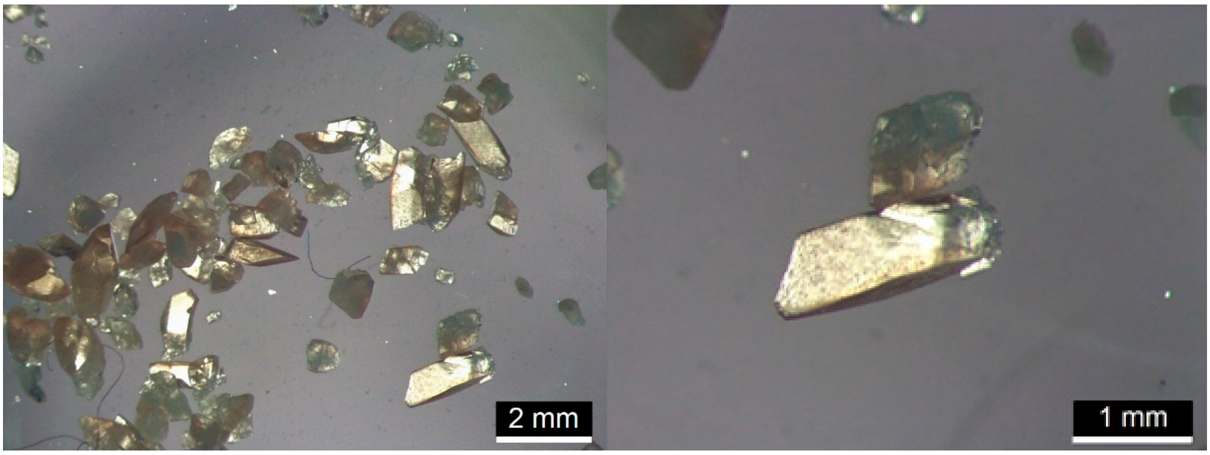

Figure S23. Crystal images of **5**.

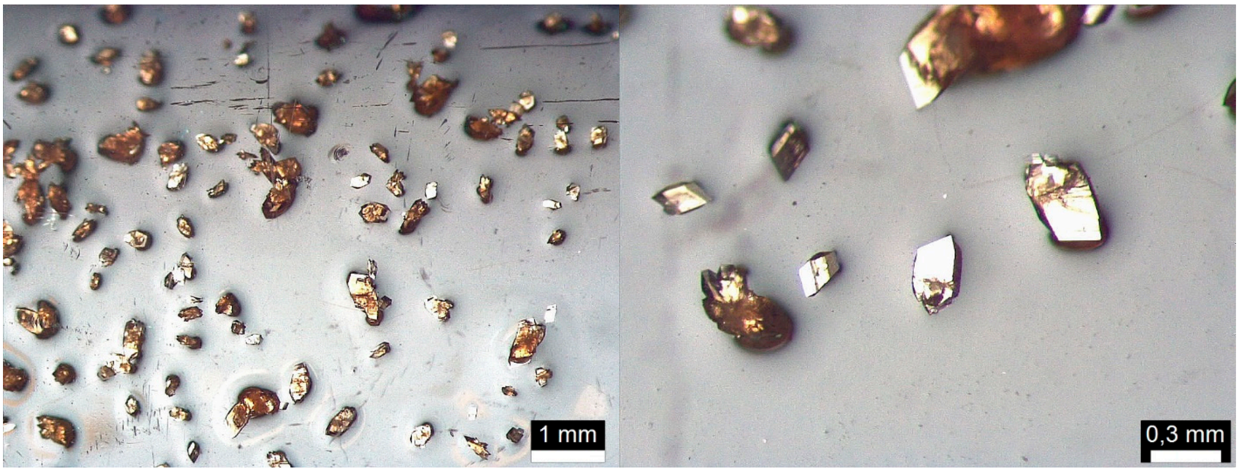

Figure S24. Crystal images of **6**.

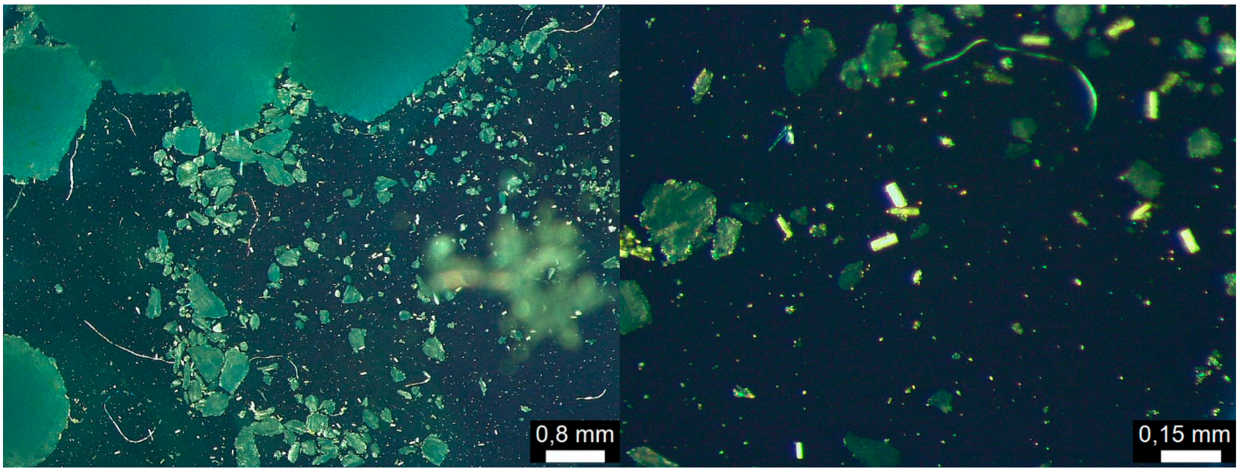

Figure S25. Crystal images of **7**.

S6. Crystal data of 1-7

The CCDC numbers 2280632-2280638 for **1**-**7** contain the supplementary crystallographic data reported in this paper. These data can be obtained free of charge from the Cambridge Crystallographic Data Centre via www.ccdc.cam.ac.uk/data_request/cif

Table S2. Crystal data for compounds **1**–**4**.

	**1**	**2**	**3**	**4**
CCDC no.	2280632	2280633	2280634	2280635
Empirical formula	$C_{24}H_{30}B_2F_8FeN_{18}$	$C_{24}H_{30}B_2F_8CoN_{18}$	$C_{24}H_{30}Cl_2O_8FeN_{18}$	$C_{24}H_{30}Cl_2O_8CoN_{18}$
M [g/mol]	800.13	803.21	825.41	828.49
T [K]	100	100	100	100
Crystal system	Trigonal	Trigonal	Trigonal	Trigonal
Space group	$P\bar{3}$	$P\bar{3}$	$P\bar{3}$	$P\bar{3}$
a [Å]	10.8198(3)	10.8024(0)	10.7561(0)	10.7538(4)
b [Å]	10.8198(3)	10.8024(0)	10.7561(0)	10.7538(4)
c [Å]	8.8597(3)	8.7938(0)	8.9294(0)	8.816(4)
α [°]	90	90	90	90
β [°]	90	90	90	90
γ [°]	120	120	120	120
V [Å^3]	898.23(6)	888.68(4)	894.66(9)	883.00(8)
Z	1	1	1	1
ϱ_{calc} [g/cm^3]	1.479	1.501	1.532	1.558
μ [mm^{-1}]	4.159	4.586	5.388	5.84
F(000)	408	409	424	425
Crystal size [mm^3]	0.07 x 0.09 x 0.10	0.24 x 0.3 x 0.44	0.23 x 0.31 x 0.31	0.11 x 0.06 x 0.03
λ (Cu K_α) [Å]	1.54184	1.54184	1.54184	1.54184
No. of unique reflections	1077	1063	1067	1059
No. of reflections	5702	10906	5526	2960
R_{int}	0.032	0.031	0.031	0.030
$R[F^2 > 2s(F^2)]$	0.0538	0.0384	0.0337	0.043
$wR(F^2)$	0.1579	0.1101	0.0949	0.115

Table S3. Crystal data for compounds **5**–**7**.

	**5**	**6**	**7**
CCDC no.	2280636	2280637	2280638
Empirical formula	$C_{18}H_{20}S_2FeN_{14}$	$C_{18}H_{20}S_2CoN_{14}$	$C_{16}H_{20}FeN_{12}Br_2(H_2O)_2{\cdot}2H_2O$
M [g/mol]	552.45	555.53	668.17
T [K]	100	100	101
Crystal system	Triclinic	Triclinic	Orthorhombic
Space group	$P\bar{1}$	$P\bar{1}$	$Fdd2$
a [Å]	8.8371(2)	8.8108(2)	30.9796(9)
b [Å]	10.3804(0)	10.3262(2)	16.4690(5)
c [Å]	13.6972(2)	13.6246(3)	9.9148(3)
α [°]	100.713(0)	100.394(2)	90
β [°]	100.685(0)	100.774(2)	90
γ [°]	97.114(0)	96.985(2)	90

V [Å^3]	1196.36(3)	1182.18(5)	5058.6(3)
Z	2	2	8
ρ_{calc} [g/cm^3]	1.534	1.563	1.755
μ [mm^{-1}]	7.020	7.68	8.91
F(000)	568	572	2699
Crystal size [mm^3]	0.09 x 0.14 x 0.47	0.09 x 0.06 x 0.05	0.09 x 0.06 x 0.05
λ (Cu K_α) [Å]	1.54184	1.54184	1.54184
No. of unique reflec-tions	4277	4218	1853
No. of reflections	21877	13910	4447
R_{int}	0.044	0.037	0.032
$R[F^2 > 2s(F^2)]$	0.0256	0.028	0.022
$wR(F^2)$	0.0662	0.063	0.049

Table S4. Selected bond lengths and bond angles for **1**–**7**.

**Compound 1**			
**Bond lengths [Å]**			
**Fe1—N1**	**2.1858(2)**	Fe1—N1^{v}	2.1858(2)
Fe1—N1^{iii}	2.1858(2)	Fe1—N1^{vi}	2.1858(2)
Fe1—N1^{iv}	2.1858(2)	Fe1—N1^{vii}	2.1858(2)
Bond angles [°]			
N1^{v}—Fe1—N1^{iv}	89.18(8)	N1^{vi}—Fe1—N1^{iii}	180.0
N1^{vii}—Fe1—N1^{iii}	89.18(8)	N1^{iv}—Fe1—N1^{vi}	89.18(8)
N1^{vi}—Fe1—N1	90.82(8)	N1^{vii}—Fe1—N1	89.18(8)
N1^{v}—Fe1—N1^{vii}	90.82(8)	N1^{vii}—Fe1—N1^{vi}	90.82(8)
N1^{iv}—Fe1—N1	90.82(8)	N1^{iii}—Fe1—N1	89.18(8)
N1^{iv}—Fe1—N1^{vii}	180.0	N1^{v}—Fe1—N1^{iii}	90.82(8)
N1^{iv}—Fe1—N1^{iii}	90.82(8)	N1^{v}—Fe1—N1	180.00(9)
N1^{v}—Fe1—N1^{vi}	89.18(8)		
Symmetry transformations: i = 1-x, 2-y, 2-z; ii = 1+x, y, 1+z; iii = -1-x+y, 1-x, z; iv = -1+y, -x+y, 1-z; v = -x, 2-y, 1-z; vi = 1+x-y, 1+x, 1-z; vii = 1-y, 2+x-y, z; viii = -x+y, 1-x, z; ix = 1-y, 1+x-y, z.			

Compound **2**			
Bond lengths [Å]			
Co1—N1	2.1490(2)	Co1—N1^{v}	2.1490(2)
Co1—N1^{iii}	2.1490(2)	Co1—N1^{vi}	2.1490(2)
Co1—N1^{iv}	2.1490(2)	Co1—N1^{vii}	2.1490(2)
Bond angles [°]			
N1^{vi}—Co1—N1^{v}	90.76(6)	N1^{vii}—Co1—N1^{iii}	90.76(6)
N1^{iv}—Co1—N1^{iii}	89.24(6)	N1^{v}—Co1—N1^{vii}	90.76(6)
N1^{iv}—Co1—N1	90.76(6)	N1^{v}—Co1—N1	180.0
N1^{vi}—Co1—N1^{iv}	90.76(6)	N1^{iv}—Co1—N1^{vii}	180.0
N1^{vi}—Co1—N1	89.24(6)	N1^{iii}—Co1—N1	90.76(6)
N1^{v}—Co1—N1^{iv}	89.24(6)	N1^{vi}—Co1—N1^{iii}	180.0
N1^{v}—Co1—N1^{iii}	89.24(6)	N1^{vii}—Co1—N1	89.24(6)
N1^{vi}—Co1—N1^{vii}	89.24(6)		
Symmetry transformations: i = 2-x, 1-y, 2-z; ii = x, -1+y, 1+z; iii = 1-x+y, 1-x, z; iv = 1-y, x-y, z; v = 2-x, 2-y, 1-z; vi = 2-y, 1+x-y, z; vii = 1-x+y, 2-x, z; viii = 1-x+y, 1-x, z; ix = 1-y, x-y, z.			

Compound **3**			
Bond lengths [Å]			
Fe1—N1	2.1909(2)	Fe1—N1^{v}	2.1909(2)
Fe1—N1^{iii}	2.1909(2)	Fe1—N1^{vi}	2.1909(2)
Fe1—N1^{iv}	2.1909(2)	Fe1—N1^{vii}	2.1909(2)
Bond angles [°]			
N1^{vi}—Fe1—N1	91.04(6)	N1^{v}—Fe1—N1^{iv}	88.96(6)
N1^{iii}—Fe1—N1^{iv}	91.04(6)	N1—Fe1—N1^{v}	180.00(6)
N1^{iii}—Fe1—N1^{vii}	88.96(6)	N1—Fe1—N1^{vii}	88.96(6)
N1^{vi}—Fe1—N1^{iii}	180.0	N1^{iii}—Fe1—N1^{v}	91.04(6)
N1^{vi}—Fe1—N1^{vii}	91.04(6)	N1^{iv}—Fe1—N1^{vii}	180.0
N1—Fe1—N1^{iii}	88.96(6)	N1^{vi}—Fe1—N1^{iv}	88.96(6)
N1—Fe1—N1^{iv}	91.04(6)	N1^{v}—Fe1—N1^{vii}	91.04(6)
N1^{vi}—Fe1—N1^{v}	88.96(6)		
Symmetry transformations: .: i = 1-x, 1-y, -z; ii = -1+x, -1+y, -1+z; iii = 2-y, 1+x-y, z; iv = 1+x-y, x, 1-z; v = 2-x, 2-y, 1-z; vi = y, 1-x+y, 1-z; vii = 1-x+y, 2-x, z; viii = 1-y, 1+x, z; ix = -x+y, 1-x, z.			

Compound **4**			
Bond lengths [Å]			
Co1—N1	2.1495(2)	Co1—N1^{v}	2.1495(2)
Co1—N1^{iii}	2.1495(2)	Co1—N1^{vi}	2.1495(2)
Co1—N1^{iv}	2.150(2)	Co1—N1^{vii}	2.150(2)
Bond angles [°]			
N1—Co1—N1^{vi}	90.88(8)	N1^{vi}—Co1—N1^{iii}	180.00(8)
N1^{iii}—Co1—N1^{vii}	90.88(8)	N1—Co1—N1^{iv}	89.12(8)
N1^{iii}—Co1—N1^{iv}	89.12(8)	N1^{v}—Co1—N1^{iii}	90.88(8)
N1^{v}—Co1—N1^{vii}	89.12(8)	N1^{vi}—Co1—N1^{v}	89.12(8)
N1^{vi}—Co1—N1^{iv}	90.88(8)	N1^{vii}—Co1—N1^{iv}	180.00(1)
N1^{v}—Co1—N1^{iv}	90.88(8)	N1^{vi}—Co1—N1^{vii}	89.12(8)
N1—Co1—N1^{iii}	89.12(8)	N1—Co1—N1^{vii}	90.88 (8)
N1—Co1—N1^{v}	180.0		
Symmetry transformations: : i = 1-x, -y, 2-z; ii =1+x, y, 1+z; iii = -y, x-y, z; iv = -x+y, -x, z; v = -x, -y, 1-z; vii = x-y, x, 1-z; vi = y, -x+y, 1-z; vii = 1-x+y, 1-x, z; ix = 1-y, x-y, z.			

Compound **5**			
Bond lengths [Å]			
Fe1—N1	2.1751(1)	Fe2—N8^{iv}	2.1732(1)
Fe1—N1^{i}	2.1751(1)	Fe2—N8	2.1732(1)
Fe1—N4	2.2014(1)	Fe2—N13^{ii}	2.1944(1)
Fe1—N4^{i}	2.2014(1)	Fe2—N13^{v}	2.1944(1)
Fe1—N7	2.1394(1)	Fe2—N14^{iv}	2.1622(1)
Fe1—N7^{i}	2.1394(1)	Fe2—N14	2.1622(1)
Bond angles [°]			
N1—Fe1—N1^{i}	180.00(1)	N8^{iv}—Fe2—N8	180.0
N1^{i}—Fe1—N4	89.08(5)	N8^{iv}—Fe2—N13^{ii}	87.13(5)
N1—Fe1—N4	90.92(5)	N8—Fe2—N13^{ii}	92.87(5)

N1—Fe1—N4^{i}	89.08(5)	N8—Fe2—N13^{v}	87.13(5)
N1^{i}—Fe1—N4^{i}	90.92(5)	N8^{iv}—Fe2—N13^{v}	92.87(5)
N4^{i}—Fe1—N4	180.0	N13^{v}—Fe2—N13^{ii}	180.00(8)
N7^{i}—Fe1—N1	89.77(5)	N14^{iv}—Fe2—N8	91.26(5)
N7^{i}—Fe1—N1^{i}	90.23(5)	N14—Fe2—N8	88.74(5)
N7—Fe1—N1^{i}	89.77(5)	N14^{iv}—Fe2—N8^{iv}	88.74(5)
N7—Fe1—N1	90.23(5)	N14—Fe2—N8^{iv}	91.26(5)
N7^{i}—Fe1—N4	90.31(5)	N14—Fe2—N13^{v}	88.97(5)
N7—Fe1—N4^{i}	90.31(5)	N14^{iv}—Fe2—N13^{ii}	88.97(5)
N7^{i}—Fe1—N4^{i}	89.69(5)	N14—Fe2—N13^{ii}	91.03(5)
N7—Fe1—N4	89.69(5)	N14^{iv}—Fe2—N13^{v}	91.03(5)
N7^{i}—Fe1—N7	180.00(6)	N14—Fe2—N14^{iv}	180.0
Symmetry transformations: i = 1-x, 1-y, 1-z; ii = 1-x, 1-y, -z; iii = 2-x, -y, 1-z; iv = 1-x, -y, -z; v = x, -1+y, z; vi = 1-x, 1-y, -z; vii = x, 1+y, z; viii = x, y, -1+z.			

Compound **6**			
Bond lengths [Å]			
Co1—N1	2.1580(2)	Co2—N8	2.1288(2)
Co1—N1^{i}	2.1580(2)	Co2—N8^{vi}	2.1288(2)
Co1—N4	2.1289(2)	Co2—N13^{iii}	2.1500(2)
Co1—N4^{i}	2.1289(2)	Co2—N13^{v}	2.1500(2)
Co1—N7	2.1105(2)	Co2—N14^{vi}	2.1304(2)
Co1—N7^{i}	2.1105(2)	Co2—N14	2.1305(2)
Bond angles [°]			
N4—Co1—N4^{i}	180.0	N13^{v}—Co2—N13^{iii}	180.0
N4—Co1—N1^{i}	89.01(6)	N8—Co2—N13^{v}	86.93(6)
N4^{i}—Co1—N1	89.01(6)	N8^{iv}—Co2—N13^{iii}	86.94(6)
N4^{i}—Co1—N1^{i}	90.99(6)	N8^{iv}—Co2—N13^{v}	93.06(6)
N4—Co1—N1	90.99(6)	N8—Co2—N13^{iii}	93.07(6)
N1—Co1—N1^{i}	180.00(6)	N8—Co2—N8^{iv}	180.00(7)
N7—Co1—N4	89.81(6)	N8—Co2—N14	91.48(6)
N7^{i}—Co1—N4	90.19(6)	N8—Co2—N14^{iv}	88.52(6)
N7^{i}—Co1—N4^{i}	89.81(6)	N8^{iv}—Co2—N14^{iv}	91.48(6)
N7—Co1—N4^{i}	90.19(6)	N8^{iv}—Co2—N14	88.52(6)
N7—Co1—N1^{i}	90.92(6)	N14^{iv}—Co2—N13^{v}	89.80(6)
N7^{i}—Co1—N1	90.92(6)	N14—Co2—N13^{iii}	89.81(6)
N7^{i}—Co1—N1^{i}	89.08(6)	N14—Co2—N13^{v}	90.19(6)
N7—Co1—N1	89.08(6)	N14^{iv}—Co2—N13^{iii}	90.20(6)
N7^{i}—Co1—N7	180.0	N14^{iv}—Co2—N14	180.0
Symmetry transformations: i = 1-x, 1-y, -z; ii = -x, 2-y, -z; iii = 1-x, 1-y, 1-z; iv = 1-x, 2-y, 1-z; v = x, 1+y, z; vi = 1-x, 2-y, 1-z; vii = 1-x, 1-y, 1-z; viii = x, -1+y, z.			

Compound **7**			
Bond lengths [Å]			
Fe1—O1	2.140(5)	Fe1—N1^{iiii}	2.177(3)
Fe1—O2	2.097(5)	Fe1—N6^{iv}	2.203(3)
Fe1—N1	2.177(3)	Fe1—N6^{ii}	2.203(3)
Bond angles [°]			
O1—Fe1—N1^{iii}	84.08(1)	O2—Fe1—N6^{iv}	86.74(1)
O1—Fe1—N1	84.08(1)	N1^{iii}—Fe1—N1	168.2(2)

O1—Fe1—N6^{iv}	93.26(1)	N1—Fe1—N6^{iv}	94.37(1)
O1—Fe1—N6^{ii}	93.26(1)	N1^{iii}—Fe1—N6^{ii}	94.37(1)
O2—Fe1—O1	180.0	N1^{iii}—Fe1—N6^{iv}	86.31(1)
O2—Fe1—N1^{iii}	95.92(1)	N1—Fe1—N6^{ii}	86.30(1)
O2—Fe1—N1	95.92(1)	N6^{ii}—Fe1—N6^{iv}	173.5(2)
O2—Fe1—N6^{ii}	86.74(1)		
Symmetry transformations: i = x, 1/2+y, 1/2+z; ii = x, -1/2+y, -1/2+z; iii = 1-x, 1-y, z; iv = 1-x, 3/2-y, -1/2+z.			

Table S5. Hydrogen-bond geometry for compounds **1**–**7**.

**Compound 1**				
D*—H···*A	***D*—H [Å]**	**H···*A* [Å]**	***D*···*A* [Å]**	*D*—H···*A* [Å]
C1—H1···F2	0.95	2.27	3.127(4)	149

Compound **2**				
D—H···*A*	*D*—H [Å]	H···*A* [Å]	*D*···*A* [Å]	*D*—H···*A* [Å]
C1—H1···N1^{iii}	0.95	2.69	3.170(3)	112
C1—H1···F1^{ix}	0.95	2.27	3.121(8)	149
C1—H1···F1A^{ix}	0.95	2.39	3.12(2)	133
C3—H3A···F2A^{x}	0.99	2.48	3.19(2)	129
Symmetry transformations: iii = y, -x+y+1, -z+1.ix= 1-y, x-y, z; x = 2-x, 1-y, 1-z				

Compound **3**				
D—H···*A*	*D*—H [Å]	H···*A* [Å]	*D*···*A* [Å]	*D*—H···*A* [Å]
C1—H1···O1	0.95	2.37	3.201(4)	146
C1—H1···O1*A*^{ix}	0.95	2.58	3.149(6)	119
C3—H3*A*···O1*A*^{viii}	0.99	2.60	3.478(7)	147
C3—H3*B*···O2*A*^{ii}	0.99	2.63	3.251(8)	121
Symmetry transformations: ii = -*x*+1, -*y*+1, -*z*+1; viii = -*y*+1, *x*-*y*+1, *z*; ix = -*x*+*y*, -*x*+1, *z*.				

Compound **4**				
D—H···*A*	*D*—H [Å]	H···*A* [Å]	*D*···*A* [Å]	*D*—H···*A* [Å]
C1—H1···N1^{vi}	0.95	2.69	3.172(3)	112
C1—H1···O1^{vi}	0.95	2.60	3.164(6)	119
C1—H1···O1*A*^{ix}	0.95	2.37	3.199(8)	146
C3—H3*A*···O2	0.99	2.65	3.265(1)	120
C3—H3*B*···O1^{ix}	0.99	2.58	3.442(8)	145
Symmetry transformations: vi = *y*, -*x*+*y*, -*z*+1; ix = -*x*+1, -*y*, -*z*+1.				

Compound **5**				
D—H···*A*	*D*—H [Å]	H···*A* [Å]	*D*···*A* [Å]	*D*—H···*A* [Å]
C1—H1···N7^{i}	0.95	2.69	3.145(2)	110
C5—H5···S2	0.95	2.93	3.7418(2)	144
C7—H7*A*···S1^{viii}	0.99	2.72	3.6888(2)	165
C7—H7*B*···S2	0.99	2.82	3.7645(2)	160
C10—H10···N11^{ix}	0.95	2.58	3.366(2)	141
C12—H12*B*···S1^{viii}	0.99	3.01	3.5452(2)	115
C15—H15*B*···S1^{x}	0.99	3.00	3.5177(2)	114
C15—H15*B*···S2^{xi}	0.99	2.74	3.5798(2)	143

C16—H16···S1^{xii}	0.95	2.71	3.5713(2)	152

Symmetry transformations: i = -*x*+1, -*y*+1, -*z*+1; viii = -*x*+2, -*y*+1, -*z*+1; ix = -*x*+2, -*y*+1, -*z*; x = -*x*+2, -*y*+2, -*z*+1; xi = *x*+1, *y*+1, *z*; xii = *x*, *y*, *z*-1.

Compound **6**

D—H···*A*	*D*—H [Å]	H···*A* [Å]	*D*···*A* [Å]	*D*—H···*A* [Å]
C1—H1···S2^{iv}	0.95	2.94	3.7500(2)	144
C11—H11···N12^{vii}	0.95	2.58	3.370(2)	141
C17—H17···S1^{viii}	0.95	2.70	3.5616(2)	152
C15—H15*A*···S2^{vii}	0.99	2.74	3.5819(2)	143
C15—H15*A*···S1^{ix}	0.99	2.98	3.5111(2)	115
C3—H3*A*···S2^{iv}	0.99	2.81	3.7601(2)	161
C3—H3*B*···S1^{x}	0.99	2.72	3.6850(2)	164
C12—H12*A*···S1^{x}	0.99	2.98	3.523(2)	115

Symmetry transformations: iv = -*x*+1, -*y*+2, -*z*+1; vii = -*x*, -*y*+1, -*z*+1; viii = *x*, *y*, *z*+1; ix = -*x*, -*y*, -*z*; x = -*x*, -*y*+1, -*z*.

Compound **7**

D—H···*A*	*D*—H [Å]	H···*A* [Å]	*D*···*A* [Å]	*D*—H···*A* [Å]
O1—H1···Br1	0.74(5)	2.58(5)	3.281(2)	159(6)
O2—H2···O3^{i}	0.77(5)	1.95(5)	2.714(4)	171(6)
C1—H1*A*···Br1^{ii}	0.95	2.86	3.721(4)	151
C2—H2*A*···N3^{v}	0.95	2.67	3.442(6)	139
C3—H3*A*···N3^{v}	0.99	2.62	3.510(5)	149
C4—H4···Br1	0.95	3.07	3.918(4)	150
C5—H5···Br1^{vi}	0.95	3.10	3.701(4)	123
C6—H6*A*···Br1^{vi}	0.99	3.13	3.818(4)	128
C8—H8···Br1^{vii}	0.95	2.83	3.737(4)	160

Symmetry transformations: i = -*x*+1, -*y*+1, *z*; ii = -*x*+1, -*y*+3/2, *z*-1/2; v = -*x*+3/4, *y*-1/4, *z*+1/4; vi = -*x*+3/4, *y*+1/4, *z*-1/4; vii = -*x*+1, -*y*+3/2, *z*+1/2.

S7. Distortion of the coordination polyhedra of 1–7

The stabilization of the LS state can depend on the distortion of the metal atom coordination sphere, such that a low distortion in the HS favors the LS state [**Error! Reference source not found.**]. Unfortunately, this behavior is different for different groups (e.g. results of octahedrally-coordinated metal atoms in nitroprussides [2] cannot be extrapolated to tetrahedrally-coordinated metal atoms in tetracyanido metal complexes due to different geometry and electronic structure [1]).

The indices for distortion were calculated using the OctaDist software [3]. The distortion parameters were calculated as follows and are listed in Table S6:

1. ζ parameter [4]: $\zeta = \sum_{i=1}^{6}|d_i - d_{mean}|$
   a. d_i: individual M–L bond distance
   b. d_{mean}: mean metal-ligand bond distance
2. Σ parameter [5]: $\Sigma = \sum_{i=1}^{12}|90 - \phi_i|$
   a. ϕ_i: individual cis angle
3. θ parameter [6]: $\theta = \sum_{i=1}^{24}|60 - \theta_i|$
   a. θ_i: individual angle between two vectors of two twisting faces

The values Σ and θ are derived by summation over the absolute values of all 12 differences 90 – ϕ_i and 24 differences 60 – θ_i, respectively, in the octahedron. The angles ϕ_i and θ_i are 90° and 60°, respectively, giving the parameters Σ and θ as zero for a perfect octahedron [1]. However, a very small deviation in ϕ_i and θ_i by only 1° will already result in Σ = 12° and θ = 24°.

The angle distortion Σ for the metal atoms in **1**–**4** and those for the metal atoms in the layers of **5** and **6** is smaller than 13°, the torsional distortion smaller than 29° (Table S6). Only for the metal atoms in the chains of **5** and **6** and in **7** is the angle and torsional distortion somewhat larger. Thus, the metal environments in **1**–**4** and those in the layers of **5** and **6** are close to a perfect octahedron.

In the literature an SCO effect is correlated to a not too high distortion.

The distortion indices in **1**–**4** are not as high as in [Fe(μ-atrz)$_3$](X)$_2$·2H$_2$O (X = BF$_4$, ClO$_4$) (atrz = *trans*-4,4′-azo-1,2,4-triazole), where SCO can be observed [7]. In case of **5** the distortion in the environment of Fe2 may be too high in comparison to [Fe(bntrz)$_3$][Pt(CN)$_4$]·H$_2$O (bntrz = 4-(benzyl)-1,2,4-triazole), a compounds where SCO is present. The parameters Σ and θ for Fe1 in **5** are, however, in a good range [8]. In [Fe$_3$(bntrz)$_6$(tcnset)$_6$] (tcnset = 1,1,3,3-tetracyano-2-thioethylpropenide) both Fe1 and Fe2 atoms give an SCO and its Fe2 atom also has distortion parameters not far from those for Fe2 in **5** [9]. Therefore, there should be more than just the distortion for the missing spin transition in **1**–**6**. Only in **7** the distortion may be too high to expect the stabilization of a low-spin state.

Table S6. Mean M–L distance and values for distortion indices of the metal atom coordination environment in compounds **1**–**7**.

Compound	M	Mean distance <D> [Å]	Distance distortion ζ [Å]	Angle distortion Σ [°]	Torsional distortion θ [°]
**1**	Fe1	2.19	0.00	9.54	21.84
**2**	Co1	2.15	0.00	9.12	20.87
**3**	Fe1	2.19	0.00	12.52	28.57
**4**	Co1	2.15	0.00	10.45	23.89
**5**	Fe1	2.17	0.13	5.84	19.49
	Fe2	2.18	0.07	20.63	56.43
**6**	Co1	2.13	0.10	8.43	23.77
	Co2	2.14	0.05	18.96	57.88
**7**	Fe1	2.17	0.19	52.89	145.78
**Comparative compounds with SCO effect**					
**$[Fe(\mu\text{-}atrz)_3](BF_4)_2\cdot 2H_2O$** [Ref. 7]	Fe1	2.18	0.04	12.83	41.30
**$[Fe(\mu\text{-}atrz)_3](ClO_4)_2\cdot 2H_2O$** [Ref. 7]	Fe1	2.18	0.03	13.13	34.13
**$[Fe(bntrz)_3][Pt(CN)_4]\cdot H_2O$**	Fe1 (HS)	2.19	–	10	23
[Ref. 8]	Fe2 (HS)	2.19	–	10	26
**$[Fe_3(bntrz)_6(tcnset)_6]$**	Fe1 (HS)	2.18	–	8	9
[Ref. 9]	Fe2(HS)	2.16	–	24	39

S8 Additional structure images

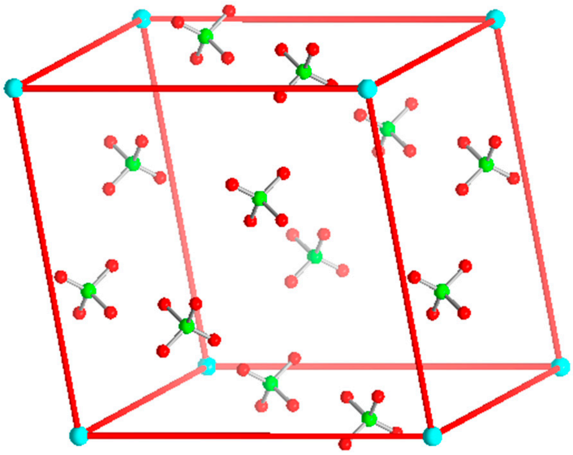

(a)

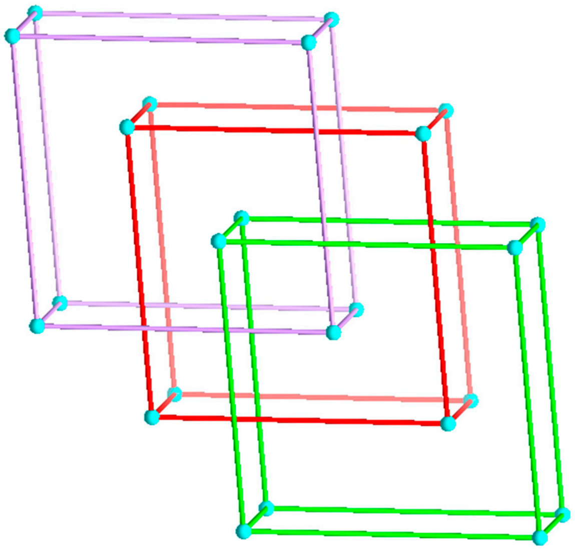

(b)

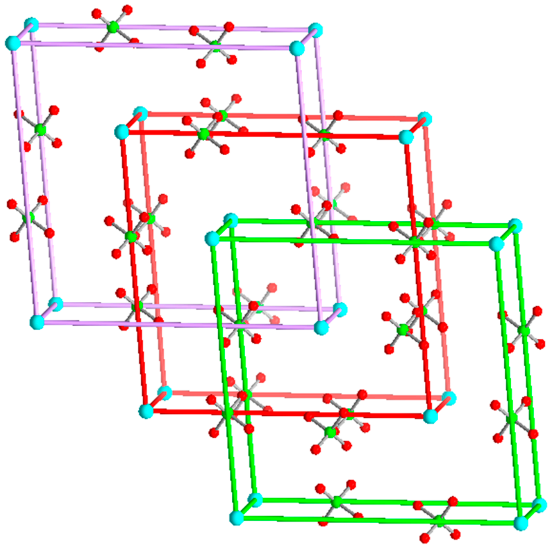

(c)

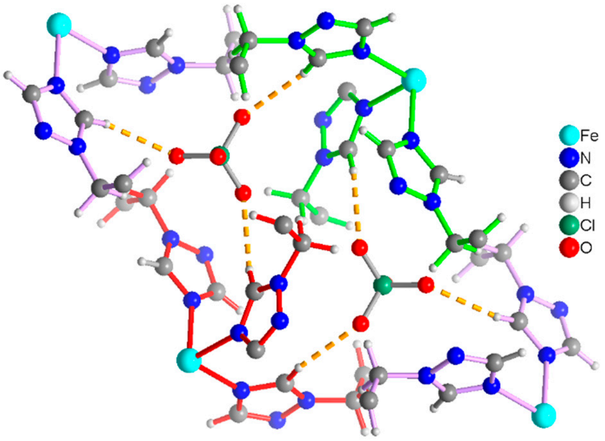

(d)

Figure S26. (**a**) Schematic presentation of the primitive lattice metal-linker topology in **3** (identical to **1, 2, 4**) with the positions of the anions on the faces of the primitive lattice. (**b**) Threefold interpenetration of three symmetry-related primitive lattices, differentiated by color. (**c**) Threefold interpenetrated lattices similar to (b) shown with ClO_4^- anions, indicating no present voids. (**d**) Template-effect of the ClO_4 (and similarly of the BF_4) anions through (triazole)C–H···O hydrogen bonds (orange dashed lines) to triazole moieties from three different networks of **3.**

S9. Powder X-ray diffraction patterns of 1-7

The increasing baseline below 5° 2Theta stems from the PXRD device measurement method with the low-background silicon holder.

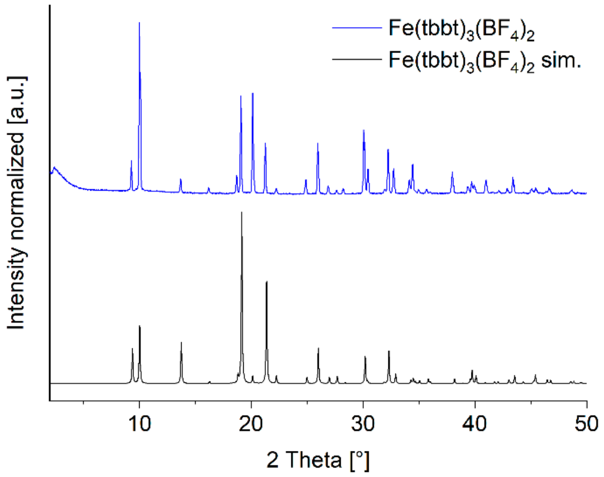

Figure S27. Comparison of diffraction pattern of **1** with its simulation.

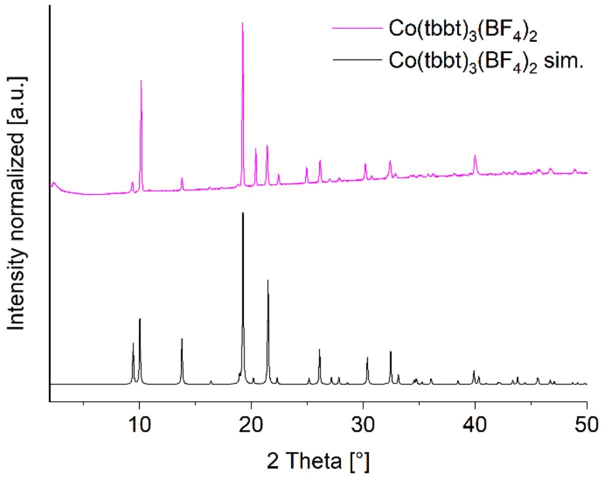

Figure S28. Comparison of diffraction pattern of **2** with its simulation.

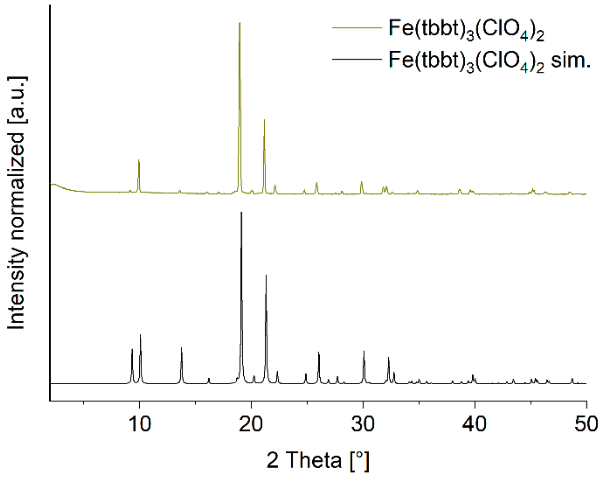

Figure S29. Comparison of diffraction pattern of **3** with its simulation.

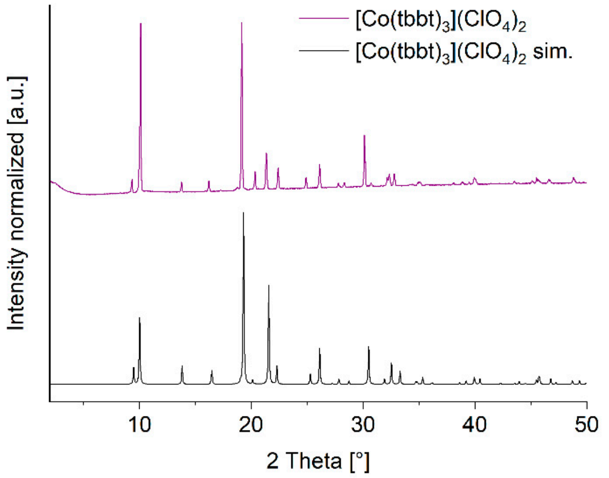

Figure S30. Comparison of diffraction pattern of **4** with its simulation.

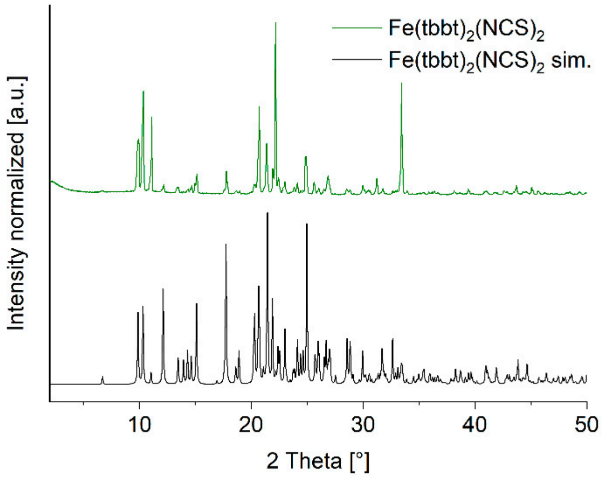

Figure S31. Comparison of diffraction pattern of **5** with its simulation.

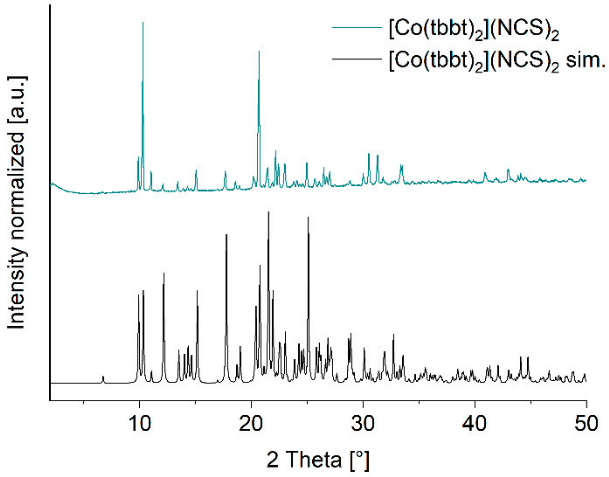

Figure S32. Comparison of diffraction pattern of **6** with its simulation.

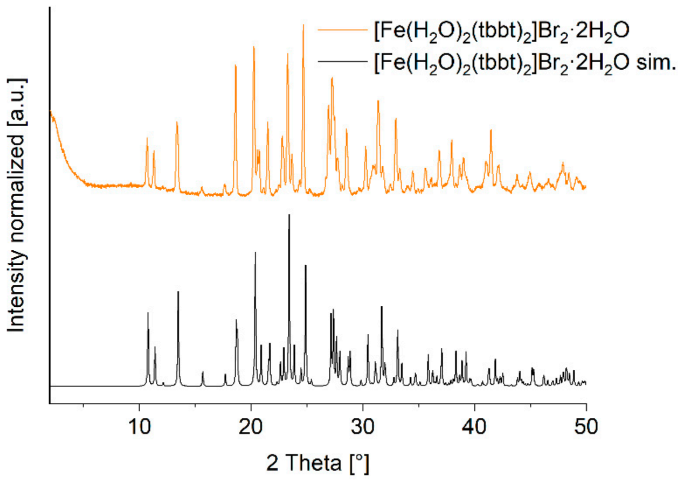

Figure S33. Comparison of diffraction pattern of **7** with its simulation.

S10. Pressure experiments of 5

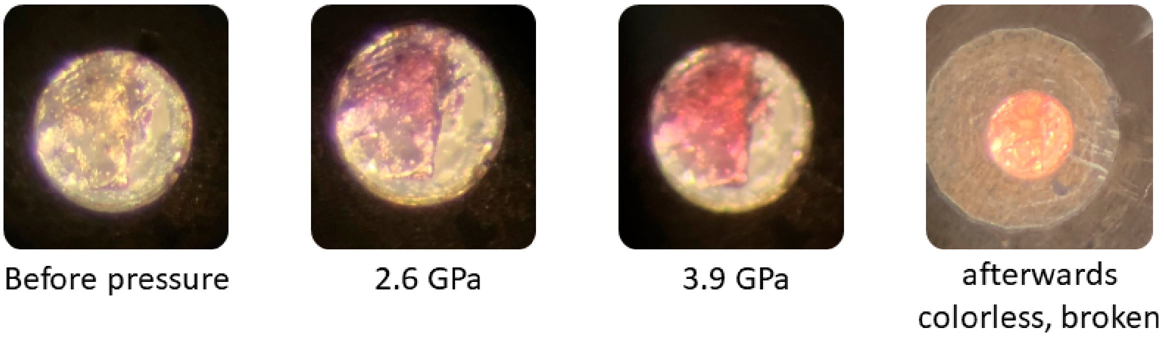

Figure S34. SC of **5** in a diamond anvil cell at different pressures.

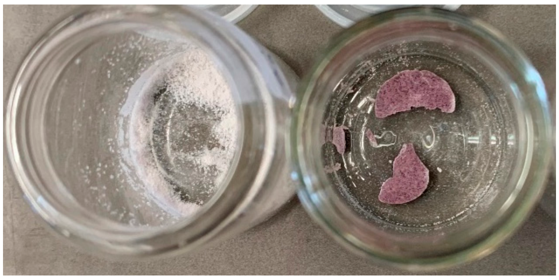

Figure S35. Powder of **5** before (left) and after (right) pressure (9 t, 10 min).

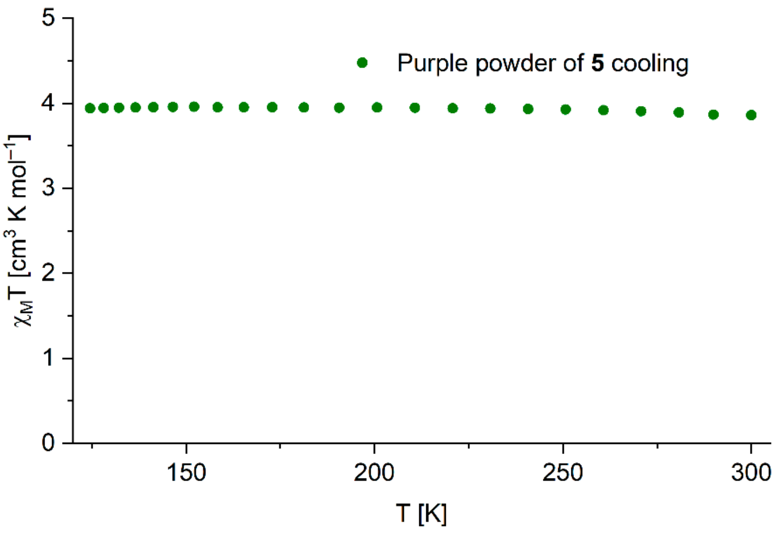

Figure S36. Magnetic properties of the purple phase of **5** measured in cooling mode. A paramagnetic ground state is present.

References

1. Avila, Y.; Pérez, O.; Sánchez, L.; Vázquez, M.C.; Mojica, R.; González, M.; Ávila, M.; Rodríguez-Hernández, J.; Reguera, E. Spin crossover in Hofmann-like coordination polymers. Effect of the axial ligand substituent and its position. *New J. Chem.* **2023**, *47*, 10781–10795. https://doi.org/10.1039/D3NJ01498C
2. Avila, Y.; Mojica, R.; Vázquez, M.C.; Sánchez, L.; González, M.; Rodríguez-Hernández, J.; Reguera, E. Spin-crossover in the Fe(4X-pyridine)$_2$ [Fe(CN)$_5$NO] series with X = Cl, Br, and I. Role of the distortion for the iron atom coordination environment. *New J. Chem.* **2022**, *47*, 238–249. https://doi.org/10.1039/D2NJ05141A
3. Ketkaew, R.; Tantirungrotechai, Y.; Harding, P.; Chastanet, G.; Guionneau, P.; Marchivie, M.; Harding, D.J. OctaDist: a tool for calculating distortion parameters in spin crossover and coordination complexes. *Dalton Trans.* **2021**, *50*, 1086–1096. https://doi.org/10.1039/d0dt03988h
4. Buron-Le Cointe, M.; Hébert, J.; Baldé, C.; Moisan, N.; Toupet, L.; Guionneau, P.; Létard, J.F.; Freysz, E.; Cailleau, H.; Collet, E. Intermolecular control of thermoswitching and photoswitching phenomena in two spin-crossover polymorphs. *Phys. Rev. B* **2012**, *85*, 064114. https://doi.org/10.1103/PhysRevB.85.064114
5. McCusker, J.K.; Rheingold, A.L.; Hendrickson, D.N. Variable-Temperature Studies of Laser-Initiated $^5T_2 \rightarrow {}^1A_1$ Intersystem Crossing in Spin-Crossover Complexes: Empirical Correlations between Activation Parameters and Ligand Structure in a Series of Polypyridyl Ferrous Complexes. *Inorg. Chem.* **1996**, *35*, 2100–2112. https://doi.org/10.1021/ic9507880
6. Marchivie, M.; Guionneau, P.; Létard, J.F.; Chasseau, D. Photo-induced spin-transition: the role of the iron(II) environment distortion. *Acta Crystallogr. B* **2005**, *61*, 25–28. https://doi.org/10.1107/S0108768104029751
7. Chuang, Y.-C.; Liu, C.-T.; Sheu, C.-F.; Ho, W.-L.; Lee, G.-H.; Wang, C.-C.; Wang, Y. New iron(II) spin crossover coordination polymers Fe(μ-atrz)$_3$X$_2$·2H$_2$O (X = ClO_4^-, BF_4^-) and Fe(μ-atrz)(μ-pyz)(NCS)$_2$·4H$_2$O with an interesting solvent effect. *Inorg. Chem.* **2012**, *51*, 4663–4671. https://doi.org/10.1021/ic202626c
8. Pittala, N.; Thétiot, F.; Triki, S.; Boukheddaden, K.; Chastanet, G.; Marchivie, M. Cooperative 1D Triazole-Based Spin Crossover Fe II Material With Exceptional Mechanical Resilience. *Chem. Mater.* **2017**, *29*, 490–494. https://doi.org/10.1021/acs.chemmater.6b04118
9. Pittala, N.; Thétiot, F.; Charles, C.; Triki, S.; Boukheddaden, K.; Chastanet, G.; Marchivie, M. An unprecedented trinuclear FeII triazole-based complex exhibiting a concerted and complete sharp spin transition above room temperature. *Chem. Commun.* **2017**, *53*, 8356–8359. https://doi.org/10.1039/c7cc04112h
